# 20(S)-protopanaxadiol prolongs lifespan and enhances stress resistance in *Caenorhabditis elegans via* the insulin/IGF-1 signaling pathway

**DOI:** 10.3389/fphar.2025.1657436

**Published:** 2025-10-14

**Authors:** Zhuo Song, Xiuci Yan, Xiaohao Xu, Tingting Lou, Yu Wang, Limei Ren, Fangbing Liu, Shuai Zhang

**Affiliations:** ^1^ The Affiliated Hospital of Jilin Medical University, Jilin Medical University, Jilin, China; ^2^ Research Center of Traditional Chinese Medicine, College of Traditional Chinese Medicine, Changchun University of Chinese Medicine, Changchun, China; ^3^ Northeast Asian Institute of Traditional Chinese Medicine, Changchun University of Chinese Medicine, Changchun, China

**Keywords:** *Caenorhabditis elegans*, 20(S)-protopanaxadiol, longevity, insulin/IGF-1 signaling pathway, DAF-16/FOXO

## Abstract

**Introduction:**

Aging is a progressive and irreversible process linked to a variety of diseases. Examination of the processes targeted by pharmacological treatments could potentially both extend lifespan and alleviate age-associated diseases. 20(S)-protopanaxadiol (20(S)-PPD), a primary ginsenoside metabolite, has many beneficial properties, although it`s anti-aging effects are unknown.

**Methods:**

Lifespan and behavioral assays were used to determine the effects of 20(S)-PPD on life span and healthy lifespan. Stress resistance was systematically determined under heat, oxidative, and chemical stress conditions. The target of 20(S)-PPD was identified by molecular docking and surface plasmon resonance. Investigation in mutant worms identified the signaling pathway and transcription factor mediating 20(S)-PPD-induced longevity.

**Results:**

20(S)-PPD could significantly extend *Caenorhabditis elegans* (*C. elegans*) lifespan without affecting food intake and reproductive output. It also improved healthspan in aging worms by ameliorating locomotor deficits and suppressing lipofuscin accumulation. Furthermore, 20(S)-PPD enhanced stress resistance and reduced age-associated reactive oxygen species (ROS) levels. Mechanistically, 20(S)-PPD bound dose-dependently to the insulin receptor (IR) with a KD value of 8.59 μM. The life-extending effects of 20(S)-PPD involved the DAF-2/insulin/IGF-1 signaling (IIS) pathway, rather than other conserved pathways. Treatment with 20(S)-PPD promoted DAF-16/FOXO activation and nuclear translocation, leading to upregulated transcription of several antioxidant and detoxification-related genes, including *lys-7*, *mtl-1*, *hsp-12.6*, *dod-3*, *sod-3*, *hsp-16.2*, *gst-4* and *sms-1*. 20(S)-PPD also upregulated the protein levels of SOD-3 and GST-4, known promoters of longevity in *C. elegans*.

**Conclusion:**

These findings demonstrate that IR is a molecular target of 20(S)-PPD and reveal a mechanism by which 20(S)-PPD promotes longevity and stress resistance, suggesting the potential of 20(S)-PPD in slowing aging and the development of age-associated disorders.

## 1 Introduction

Aging is a progressive and irreversible process accompanied by an overall deterioration in functioning (López-Otín et al., 2013). Aging is also linked to the development of various disorders and diseases, such as cardiovascular disorders, neurodegeneration, and cancer (Guo et al., 2022; Hou et al., 2019; López-Otín et al., 2023; North and Sinclair, 2012). The examination of the molecular factors underlying aging could lead to the development of anti-aging interventions for the promotion of longer and healthier lives. There is extensive evidence of the effectiveness of traditional Chinese medicine and its associated components in combating aging and age-related deterioration (Lin et al., 2025). 20(S)-protopanaxadiol (20(S)-PPD), a product of ginsenoside hydrolysis and deglycosylation enhanced by gut microbes, has been shown to possess numerous biological activities, and is effective in treating inflammation, fatigue, tumors, and diabetes (Oh et al., 2015; Pan et al., 2018; Song et al., 2023; Zhang et al., 2017). However, the functions of 20(S)-PPD in delaying aging and the underlying processes involved remain unknown.

Using humans as a study model for aging is challenging due to ethical concerns, extended lifespan, and difficulties in controlling variables. Research on different organism models, from yeast to mammals, has demonstrated that the mechanisms controlling lifespan are evolutionarily conserved (Holtze et al., 2021; Taormina et al., 2019). *Caenorhabditis elegans* (*C. elegans*) provides numerous benefits for studying anti-aging agents due to its short lifespan (about 4 weeks) and ease of genetic manipulation, making *C. elegans* a widely accepted model in aging research (Nigon and Félix, 2017). Various pathways involved in longevity have been characterized in *C. elegans*, including the insulin/IGF-1 signaling (IIS), mammalian target of rapamycin (mTOR), germline, mitochondrial respiratory chain, and dietary restriction pathways (Gruber et al., 2015). Moreover, healthspan, which is a significant measure of aging, may also be evaluated in *C. elegans* by examining age-related physiological processes (Gruber et al., 2015). Thus, *C. elegans* has been used in investigations on drug screening, as well as in basic research related to anti-aging mechanisms.

The IIS pathway was initially identified as an aging-related pathway (Kenyon et al., 1993; Zhang et al., 2022). Research using mutations of the insulin receptor (IR) has demonstrated its involvement in promoting longevity in several species, including *C. elegans*, *Drosophila* and mice (Kenyon et al., 1993). The *daf-2* gene, encoding the only IR/IGF-1R homolog in *C. elegans*, is strongly conserved, and *daf-16* is homologous to the mammalian fork head box O (FOXO) transcription factor FOXO3A (Zečić and Braeckman, 2020). The *daf-2*/*daf-16* axis in *C. elegans* includes AKT-1/2, PDK-1, AGE-1/PI3-K and DAF-16/FOXO (Sun et al., 2017). The IIS cascade, involving DAF-2 to AKT-1/2, is linked to shorter lifespan through inhibition of DAF-16, restricting its transcription factor activity (Zečić and Braeckman, 2020). It has been found that loss-of-function mutations in kinases upstream of DAF-16 can extend longevity (Murtaza et al., 2017). Dephosphorylation of DAF-16 leads to its activation and subsequent nuclear translocation, where it promotes the transcription of genes associated with antioxidant and detoxification processes, enhancing the lifespan and healthspan of *C. elegans* (Murtaza et al., 2017).

Here, the effects of 20(S)-PPD in the modulation of aging were investigated in *C. elegans*. The results suggested that 20(S)-PPD not only prolonged lifespan and healthspan but also enhanced tolerance to multiple stressors and reduced ROS levels. 20(S)-PPD was found to bind to IR *in vitro*. Moreover, 20(S)-PPD-mediated longevity extension was dependent on the IIS pathway, thereby inducing the nuclear translocation of DAF-16/FOXO to enhance transcription of target genes. These results demonstrate that IR is a previously unrecognized target of 20(S)-PPD and show the effectiveness of 20(S)-PPD in alleviating the effects of aging, suggesting its potential in preventing and treating aging and age-related conditions.

## 2 Materials and methods

### 2.1 Materials

20(S)-protopanaxadiol (20(S)-PPD) (CAS: 30636-90-9) was obtained from Yuanye Bio-Technology (Shanghai, China). Tert-butyl hydroperoxide (tBHP), CM-H_2_DCFDA, dimethyl sulfoxide (DMSO) and paraquat (PQ) were purchased from Sigma-Aldrich (St. Louis, MO, United States). Human IR Protein expressed by HEK293 with C-His labeled tag was purchased from MCE (Trenton, NJ, United States). Levamisole and dihydroethidium (DHE) were acquired from Thermo Scientific (Waltham, MA, United States). Isoamyl alcohol and other reagents were purchased from BICR (Beijing, China).

### 2.2 *Caenorhabditis elegans* strains and cultivation

The worms were cultivated at 20 °C on agar plates with Nematode Growth Medium (NGM) and a bacterial lawn of *Escherichia coli* (*E. coli*) OP50 (Stiernagle, 2006). Fresh worms stocks were set up from worms frozen in glycerol at −80 °C to prevent genetic drift. The strains employed in this investigation were provided from the *Caenorhabditis* Genetics Center (CGC, University of Minnesota, Minneapolis, MN, United States) and included N2/Bristol, wild-type; AM44, *rmIs190 [F25B3.3p::Q67::CFP]*; CB1368, *daf-2(e1368) III*; CF1553, *muIs84 [(pAD76) sod-3p::GFP + rol-6(su1006)]*; DA1116, *eat-2(ad1116) II*; MQ887, *isp-1(qm150) IV*; CB1370, *daf-2(e1370) III*; TJ356, *zIs356[daf-16::gfp] IV*; CF1308, *daf-16(mu86) I*; CB4037, *glp-1(e2141) III*; CL2166, *dvIs19[(pAF15) gst-4p::GFP::NLS] III*. Mutants were subjected to three backcrosses with wild-type (WT) strains prior to use. Before the analyses, all worms were housed at a temperature suitable for their normal growth and were raised for at least two generations with access to food for their optimal health.

According to the Wormbook protocol (https://wormbook.org), *daf-2(e1370)* was crossed with *daf-16(mu86)* to generate the double mutant *daf-2(e1370);daf-16(mu86)*. Briefly, approximately 5,000 *daf-16(mu86)* mutants at the late L4 stage were heat-shocked at 30 °C for 4–6 h to increase male numbers (minimum 5–10). These male mutants were mated with *daf-2(e1370)* hermaphrodites at the L4 stage. Double mutants were constructed following standard procedures up to the F2 generation and were confirmed by PCR genotyping and DNA sequencing (Rogers et al., 2008). Primer sequences used in the crossing experiment are provided in Supplementary Table S1.

### 2.3 Lifespan assessments

According to the standard protocols described in a previous study, the 20(S)-PPD was freshly added from a 400 mM stock solution into the NGM media before being poured onto the plates. The control population was treated with DMSO, and 200 μL *E coli* OP50 suspension was seeded on the 6-cm-diameter NGM plates. Inactivated OP50 was prepared by UV irradiation at 254 nm, and 200 µL of a 5× concentrated inactivated OP50 suspension was seeded onto 6-cm-diameter NGM plates (Wang et al., 2021). Worm lifespan was assessed at 20 °C as previously described (Chen et al., 2020). Briefly, growth-stage-synchronized worms were grown as described above in the presence of 100, 200, or 400 μM 20(S)-PPD or the control solution (DMSO) from eggs to death. At least 30 worms per group were investigated each day and were recorded as dead if they showed no response to prodding with a platinum wire probe. Worms were placed on fresh plates each day, with the number of dead worms recorded. Survival rates were measured until death and curves were drawn. All lifespan assessments were performed three times independently. The detailed statistics of the lifespan assays are presented in Supplementary Table S2.

### 2.4 Bacterial avoidance assay

To assess the preference of the worms toward OP50 (DMSO alone) and OP50 combined with 20(S)-PPD (100, 200, and 400 μM), approximately 50 worms were placed in the center of the plate. After 3 and 6 h, worms were counted on each side. All bacterial avoidance assays were repeated three times independently.

### 2.5 Bacterial growth rates

Experiments were performed as described previously (Wang et al., 2021). Overnight cultures of *E. coli* OP50 were diluted 1: 1000 and mixed with various concentrations of 20(S)-PPD in 96-well plates. Cultures were shaken at 37 °C, and absorbance at 595 nm was measured hourly for at least 8 h using a microplate reader (Infinite M200 Pro, Tecan, Männedorf, Switzerland). Each condition included five replicates, and the experiment was repeated twice.

### 2.6 Locomotion and pumping rate assay

Growth-stage-synchronized N2 worms were cultivated in the presence of 100, 200, or 400 μM 20(S)-PPD or control solution (0.1% DMSO) from the egg stage until the end of the experiment. Locomotory ability was assessed on day 7 and 11. In the center of an NGM plate without *E. coli* OP50, a coordinate system was established with the longitudinal axis of the pharyngeal pump as the X-axis and the perpendicular direction as the Y-axis (Zheng et al., 2022). A complete sinusoidal waveform displacement of the worm was counted as one effective body bend. Synchronized worms were placed on a plate containing a drop of M9 buffer and allowed to adapt for 30 s. The number of effective body bends during locomotion over 30 s was recorded. Each assay was performed at least twice, with 20 worms scored per group. Pharyngeal pumping was evaluated on day 7 and 11 of adulthood by recording the number of pharyngeal contractions within 30 s (Wang et al., 2021). For each group, 20 worms were scored.

### 2.7 Egg-laying assay

Growth-stage-synchronized N2 worms were grown on plates containing 100, 200, and 400 μM 20(S)-PPD or the control solution (DMSO alone) from eggs until the end of the experiment. Twenty worms from each group were then placed on individual plates and the numbers of eggs produced were counted manually.

### 2.8 Lipofuscin assay

Growth-stage-synchronized N2 worms were grown with 100, 200, and 400 μM 20(S)-PPD or the control solution (DMSO alone) from eggs until the end of the experiment. The accumulation of lipofuscin, a pigment associated with aging and cellular damage (cellular wear and tear), was examined as described (Chen et al., 2017). Lipofuscin autofluorescence in the intestines was evaluated and imaged in 7 and 11-day-old adult worms using a Lionheart Fx Automated Live Cell Imager (BioTek, VT, United States) through the use of blue excitation light (405-488 nm) (with a magnification to 10 times). The quantification of the fluorescence intensity (20 worms per group) was quantified using ImageJ software (Wayne Rasband, NIH, UnitedStates).

### 2.9 Oxidative stress assay

For oxidative stress resistance, worms were treated with 100, 200, or 400 μM 20(S)-PPD from egg stage to day 1, while control cohorts received 0.1% DMSO. Day 1 worms were transferred to NGM plates containing 9.125 mM tBHP to induce ROS accumulation (Li et al., 2019). Approximately 45 worms per group were incubated at 20 °C, and mortality was recorded hourly until all worms had died. Worms that did not respond to prodding with a platinum wire probe were considered dead. Survival curves were generated, and assays were repeated three times independently. Furthermore, 1-day-old worms were exposed to 200 mM paraquat (PQ) for 4 h, and mortality was quantified as described previously (approximately 100 worms per group, three independent replicates) (Huang et al., 2025).

### 2.10 Heat stress assays

Worms were pretreated with 100, 200, or 400 μM 20(S)-PPD from egg stage to day 1; controls received 0.1% DMSO. After pretreatment, approximately 45 worms per group were transferred to fresh NGM plates and subjected to lethal heat stress at 35 °C. Survival was monitored every 2 h by prodding with a platinum wire probe until complete cohort mortality. All assays were repeated three times independently.

### 2.11 Detection of intracellular ROS

Twenty 11-day-old worms, which were grown in the presence or absence of 20(S)-PPD, were washed 3 times with M9 buffer. The worms were then incubated in 5 μM fluorescent DHE, in M9 buffer in the dark at 20 °C for 1 h. After washing, the worms were mounted on a glass slide, paralyzed with 10 µL levamisole (10 µM in PBS), and the total fluorescence of each worm was analyzed by fluorescence microscopy, where that the intensity of fluorescence was dependent on intracellular oxidation of DHE by superoxide/ROS to form 2-hydroxyethidium (2-EOH) (Jiang et al., 2022). Representative images at ×10 magnification were taken by a Lionheart Fx Automated Live Cell Imager (BioTek, VT, United States) with red excitation light (530-620 nm). Likewise, Twenty 2-day-old worms treated by 5.5 mM tBHP were washed 3 times with M9 buffer, and 20 µM fluorescent dye CM-H_2_DCFDA was incubated for 30 min at 20 °C in the dark (Wang et al., 2021). The worms were washed once with M9 buffer to remove excess dye and then mounted on a glass slide with a drop levamisole for paralysis. Representative images at ×10 magnification were taken with excitation at 485 nm and emission at 520 nm. The fluorescence intensity (20 worms per group) was analyzed by using ImageJ (National Institutes of Health, Bethesda, MD, United States).

### 2.12 Molecular docking technology

Molecular docking was performed as previously described. The structure of IR was retrieved from the Protein Data Bank (https://www.rcsb.org/), while the 2D structure of 20(S)-PPD was obtained from PubChem (https://pubchem.ncbi.nlm.nih.gov/). Removal of water molecules from the IR structure was performed with PyMOL 2.3.0, and the hydrogen bonds and charge were determined using AutoDocktools (v1.5.6). Docking between IR and 20(S)-PPD was under taken with AutoDockVina1.1.2.

### 2.13 Surface plasmon resonance (SPR)

SPR was performed using a Biacore T200 instrument (Cytiva, Washington DC, United States). The IR protein was diluted 20-fold with a fixation reagent (10 mM sodium acetate, pH 4.0), followed by immobilization on a sensor chip utilizing standard amine coupling. Varying concentrations of 20(S)-PPD in the running buffer were tested for binding to IR proteins. The results were analyzed using Biacore T200 Evaluation Software, with data fitting using an affinity 1:1 binding model.

### 2.14 RNA isolation and RT-qPCR

Total RNA was isolated from the worms using TRIzol (Tiangen, Beijing, China) as directed, followed by assessment of the concentration using a NanoDrop 2000/c spectrophotometer (Thermo Fisher Scientific, Waltham, MA, United States). RT-qPCR was performed using SYBR Green PCR Master Mix (Bio-Rad, Hercules, CA, United States) and a Bio-Rad CFX96 RealTime PCR Detection System. The endogenous control was *act-1*. The 2^−ΔΔCt^ protocol was adopted for assessing the relative mRNA expression levels and is indicated as the mean fold change of 3 separate tests. Supplementary Table S3 provides details of the primer sequences.

### 2.15 Nuclear localization of DAF-16

TJ356, a reporter strain of *C. elegans* harboring the DAF-16-GFP fusion protein, was grown as described above with 100, 200, and 400 μM 20(S)-PPD or the control solution (DMSO alone) from eggs until young adulthood. To paralyze the worms, 100 worms from each group were placed on an agarose pad (2%) on a glass slide, and a drop (approximately 10 μL) of 10 μM levamisole was added. Images of GFP fluorescence ×20 magnification were taken using an Olympus FV-1000 confocal microscope (Olympus, Tokyo, Japan) with excitation at 485 nm and emission at 520 nm. For the scoring of nuclear localization, the worms were classified into three categories based on their fluorescence intensity: intermediate, cytoplasmic, and nuclear (Su et al., 2018). The percentage of DAF-16 nuclear localization in each group was evaluated by dividing the value of the group by the total value. All assessments were performed three times independently.

### 2.16 SOD-3:GFP and GST-4:GFP expression

The CL2166 and CF1553 reporter strains were employed to visualize GST-4 and SOD-3 levels, respectively. L4 larvae of similar age were kept in NGM agar dishes with *E. coli* OP50 and 20(S)-PPD (200 μM). After 3 days, worms were transferred to an agarose pad (2%) and a drop of the paralyzing agent levamisole (10 µM in PBS) was introduced. Images at ×10 magnification were taken by a Lionheart Fx Automated Live Cell Imager (BioTek, VT, United States) with excitation at 485 nm and emission at 520 nm. Subsequently, the fluorescence intensity representing the levels of GST-4 and SOD-3 (20 worms per group) was determined by quantification of the GFP fluorescence intensity using ImageJ.

### 2.17 Aversive olfactory conditioning chemotaxis assay

**FIGURE 1:**
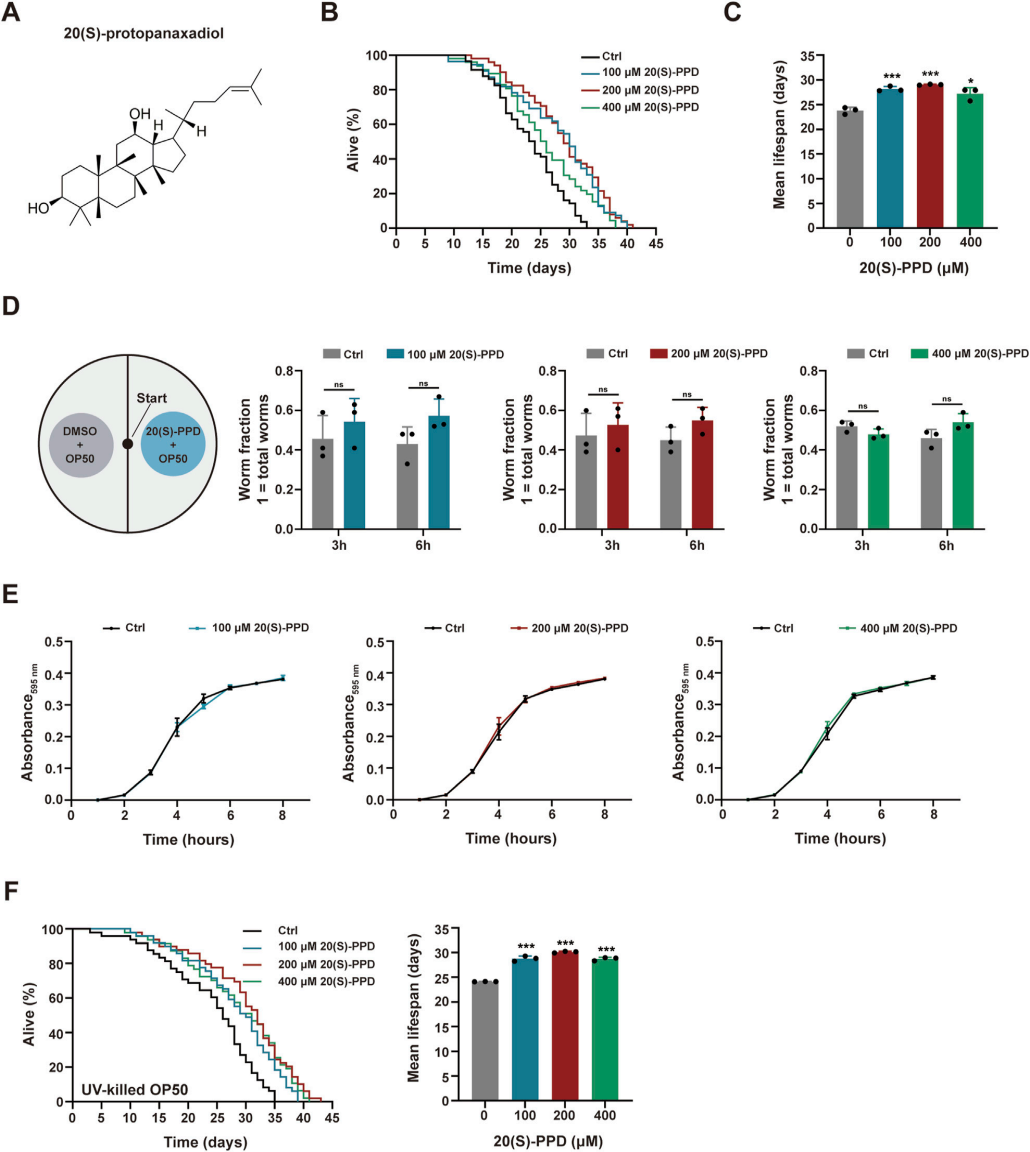
20(S)-PPD extends lifespan of (C) *elegans.* The strains used were: N2, wild type*.* Ctrl, treated with DMSO. 20(S)-PPD, treated at concentrations of 100, 200, and 400 μM 20(S)-PPD, separately. **(A)** The chemical structure of 20(S)-PPD. **(B,C)** Lifespan assays for N2 treated with concentrations of 100, 200, and 400 μM 20(S)-PPD, separately. For each group, worms were counted as detailed in the methods (n > 30). Survival plot **(B)** and average lifespan **(C)** of each group (n = 3). **(D)** A bacterial avoidance assay was performed using 20(S)-PPD treated-*Escherichia coli* OP50 bacteria. The N2 worms (L4 larvae n = 50) were transferred to the NGM plate’s center, where the left side bacteria contained 100, 200, and 400 μM 20(S)-PPD dissolved in *Escherichia coli* OP50 bacteria, whereas those on the right side did not (Ctrl). The number of worms on each side of the agar plate was evaluated by picking and counting them after 3 and 6 h. The ratio of the number of worms crawling on each side of the plate indicated the worm fraction (n = 3). **(E)** Treatment with 20(S)-PPD at 100, 200, and 400 μM for 8 h made no difference on the growth of OP50 bacteria compared to the Ctrl group. **(F)** Lifespan assays for N2 treated with concentrations of 100, 200, and 400 μM 20(S)-PPD feeding with UV-killed *Escherichia coli* OP50 bacteria, separately. For each group, worms were counted as detailed in the methods (n > 30). Survival plot (left) and average lifespan (right) of each group (n = 3). Statistical analysis of the lifespan was performed using GraphPad 9 and *p* values were calculated by the log-rank test. Numerical data were analyzed by Student’s t-test and values were presented as mean ± SD. ns, no significance; statistical significance is indicated as *p < 0.05, **p < 0.01, and ***p < 0.001 compared to the Ctrl group.

Chemotaxis to volatile compounds was performed on 9-cm NGM plates using day 1, 7, and 11 adult worms, as described previously (Fang et al., 2019) (Figure 1H). Briefly, 5 μL of pure isoamyl alcohol was placed on the lid of a conditioning non-bacterially seeded NGM plate, and worms were conditioned to isoamyl alcohol for 90 min. Both naive and conditioned animals were subsequently exposed to isoamyl alcohol for 90 min (gradient sources: isoamyl alcohol, 1: 100 dilution in DMSO). Drip positions of 2 μL isoamyl alcohol and DMSO are shown in Figure 1H. Approximately 100 adults per group were scored per assay. The chemotaxis index (CI) was calculated as the number of animals at the chemical source minus those in the trap, divided by the total number of animals. CI is negatively correlated with cognitive ability. All assays were performed at least three times independently.

### 2.18 Statistical analysis

Data are shown as mean ± SD. Distribution normality and variance were assessed, and data were analyzed using independent-samples two-tailed *t*-tests or ANOVAs, as appropriate. Survival data were compared with log-rank (Mantel-Cox) tests. GraphPad Prism 9 was employed for all other statistical tests. *p* < 0.05 was deemed significant.

## 3 Results

### 3.1 20(S)-PPD extends lifespan and improves healthspan in *Caenorhabditis elegans*

**FIGURE 2:**
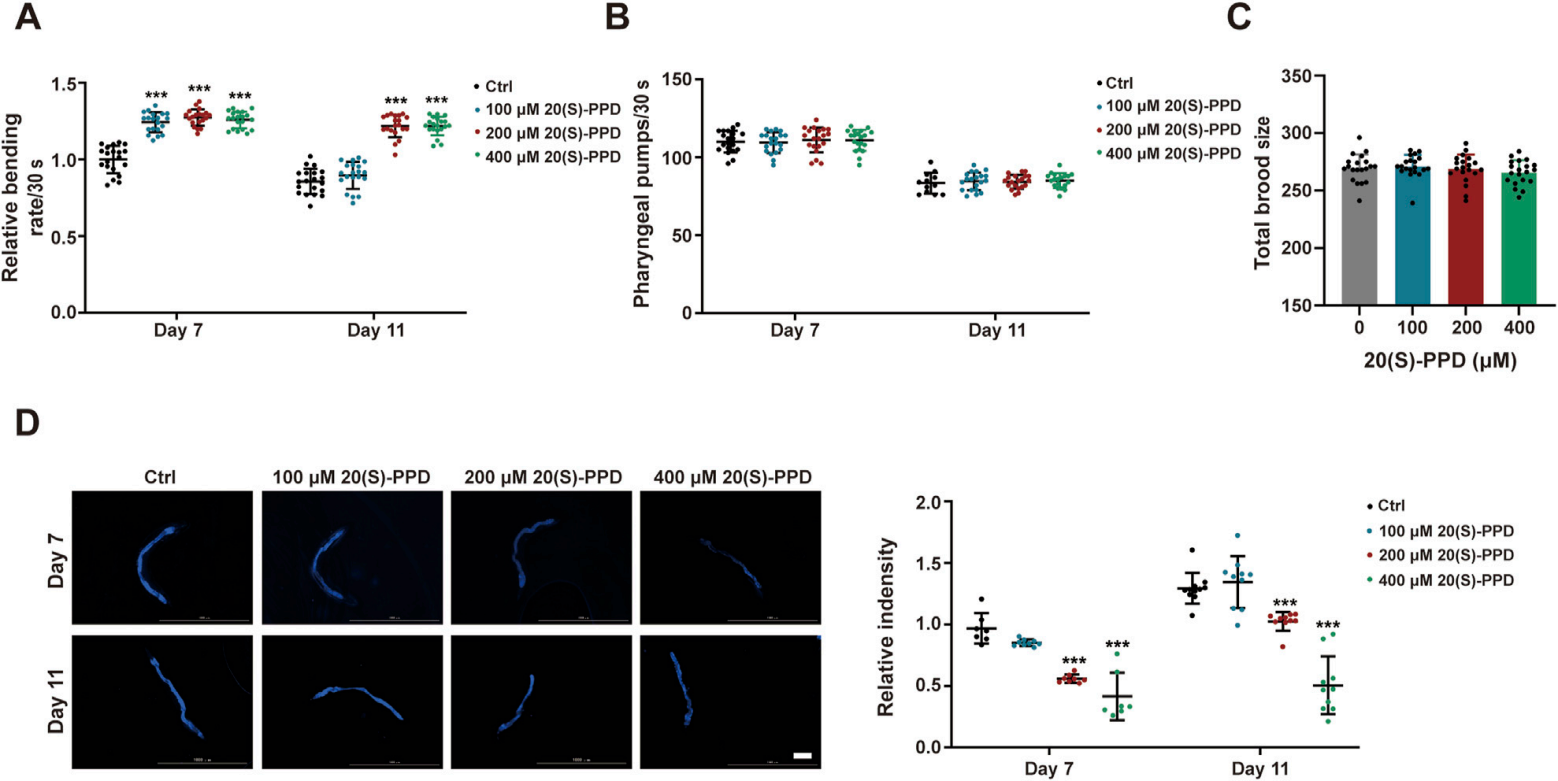
20(S)-PPD extends the healthspan of (C) *elegans.* The strains used were: N2, wild type*.* Ctrl, treated with DMSO. 20(S)-PPD, treated at concentrations of 100, 200, and 400 μM 20(S)-PPD, separately. **(A)** Locomotion as determined by the number of the effective body bends per 30 s in 100, 200, and 400 μM 20(S)-PPD-treated worms (n = 20) at day 7 and day 11. **(B)** The pharyngeal pumping rates of worms treated with 100, 200, and 400 μM 20(S)-PPD (n = 20). The pumping rates within 30 s were recorded at day 7 and day 11. **(C)** The fertility is determined by counting the number of eggs per worm (n = 20). **(D)** At day 7 and 11 after 20(S)-PPD treatment, blue autofluorescence, representing lipofuscin accumulation, was detected by fluorescence microscopy. The relative fluorescence intensity was evaluated by using ImageJ software, and the fluorescence of the untreated nematodes was set as 1 (n ≥ 10). The scale bar indicates 0.2 mm. Statistical analysis of the lifespan was performed using GraphPad 9 and *p* values were calculated by the log-rank test. Numerical data were analyzed by Student’s t-test and values were presented as mean ± SD. ns, no significance; statistical significance is indicated as *p < 0.05, **p < 0.01, and ***p < 0.001 compared to the Ctrl group.

To evaluate the effects of 20(S)-PPD on *C. elegans* longevity, growth-synchronized WT worms were treated with varying amounts of 20(S)-PPD (100, 200, and 400 μM) from eggs until death. Marked increases in worm survival were observed after 20(S)-PPD treatment, with lifespan extensions of 18.42, 22.08, and 14.30% at concentrations of 100, 200, and 400 μM, respectively (Figures 1A–C), indicating a dose-dependent effect of 20(S)-PPD on lifespan with peak efficacy at 200 μM. Since diet restriction has a regulatory impact on longevity in *C. elegans*, an avoidance assay was performed to eliminate the possibility that *C. elegans* preferred OP50 bacteria treated with 20(S)-PPD as food. As depicted in Figures 1C,D *elegans* showed no preference for food, indicating that it did not prefer or avoid *E. coli* OP50 bacteria treated with 20(S)-PPD. To exclude the possibility that 20(S)-PPD might affect *C. elegans* lifespan indirectly by inhibiting OP50 growth, we first measured the development of the *E. coli* strain OP50 following treatment with 100, 200, and 400 μM 20(S)-PPD. No effect on bacterial growth was observed at any concentration (Figure 1E). Furthermore, following standard protocols (Wang et al., 2021), worms were fed UV-inactivated OP50 to eliminate the influence of bacterial viability on 20(S)-PPD-induced lifespan extension. As shown in Figure 1F, the lifespan of worms fed inactivated OP50 was consistent with that of worms fed active OP50; all concentrations of 20(S)-PPD extended worm lifespan by 19.41, 24.80, and 19.04% at 100, 200, and 400 μM, respectively. These results indicate that 20(S)-PPD can directly affect the longevity of *C. elegans* in a dose-dependent manner. As reduced locomotion, neurological deterioration, reduced fertility, and increased lipofuscin levels are known changes associated with aging in *C. elegans* (Shen et al., 2018), these factors were assessed to elucidate the potential health benefits of 20(S)-PPD. Motility, an indicator of muscle function and overall health, was quantified by counting the number of effective body bends within 30 s, following previously established methods (Zheng et al., 2022). As shown in Figure 2A, treatment with 200 and 400 μM 20(S)-PPD significantly increased the frequency of effective body bends in both 7- and 11-day-old N2 worms, indicating that 20(S)-PPD improved motility of *C. elegans*. In addition, the rate of pharyngeal pumping did not change in N2 worms after treatment with 20(S)-PPD (Figure 2B). As extended lifespan is often linked to reduced fertility, the number of eggs and length of development in N2 worms were assessed after treatment with 20(S)-PPD. The results of the brood sizes suggested that worm fertility did not change after exposure to 20(S)-PPD relative to the controls (Figure 2C), indicating that 20(S)-PPD did not prolong lifespan by decreasing fertility and egg numbers in N2 worms.

Lipofuscin, a pigment associated with aging, gradually accumulates during the aging process, impairing cellular adaptability and contributing to age-associated disorders such as cardiovascular disease, neurological disorders, and macular degeneration (Terman and Brunk, 2004). In *C. elegans*, intestinal lipofuscin shows autofluorescence detectable under a fluorescence microscope (Vora et al., 2013). Therefore, we measured autofluorescence to assess the effect of 20(S)-PPD on lipofuscin accumulation in worms. Relative to the autofluorescence seen the control worms, all 20(S)-PPD doses inhibited the accumulation of intestine lipofuscin in 7-day-old adults, while 200 and 400 μM 20(S)-PPD reduced lipofuscin accumulation in 11-day-old adults (Figure 2D). These findings indicate that 20(S)-PPD both prolongs lifespan and induces health benefits in *C. elegans*.

### 3.2 20(S)-PPD improves stress tolerance and blocks ROS accumulation in *Caenorhabditis elegans*

**FIGURE 3:**
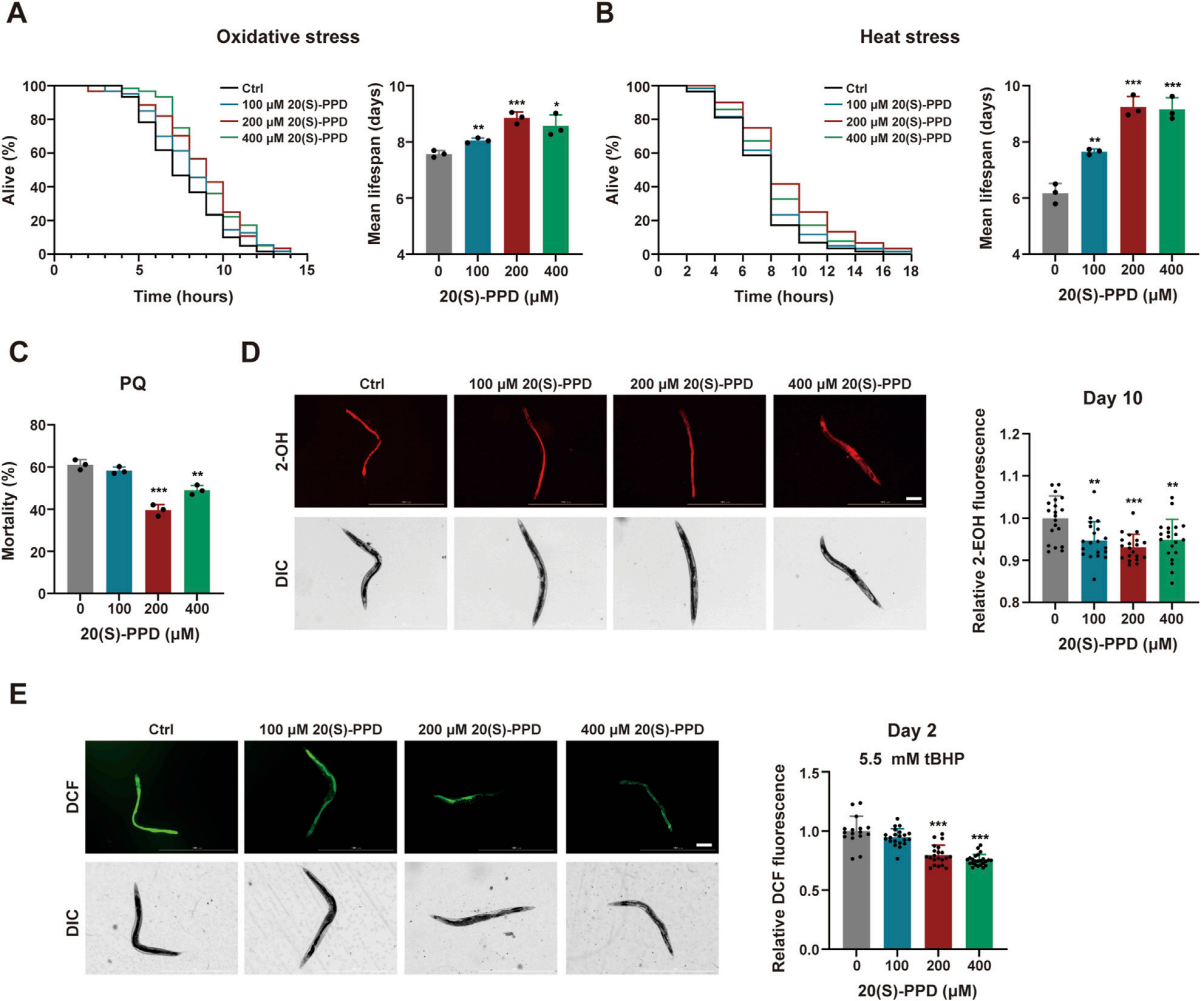
20(S)-PPD enhances stress resistance and decreases the accumulation of ROS in (C) *elegans*. The strains used were: N2, wild type*.* Ctrl, treated with DMSO. 20(S)-PPD, treated at concentrations of 100, 200, and 400 μM 20(S)-PPD, separately. **(A)** Survival curves and mean lifespan of N2 worms exposed to 9.125 mM tBHP for oxidative stress analysis (n ≥ 45 worms for each group each time, experiment repeated 3 times). **(B)** Survival curves of N2 worms under 35 °C for heat stress analysis (n ≥ 45 worms for each group each time, experiment repeated 3 times). **(C)** The resistance to oxidative stress of N2 worms to 200 mM PQ (n = 50 worms for each group each time, experiment repeated 3 times). **(D)** 10-day-old 20 adult worms each group were incubated in 5 μM DHE and the total fluorescence of each worm was analyzed by fluorescence microscopy, where that the intensity of fluorescence was dependent on intracellular oxidation of DHE by superoxide/ROS to form 2-EOH. The fluorescence intensity was analyzed with ImageJ. The scale bar indicates 0.2 mm. **(E)** 2-day-old 20 adult worms each group treated with 5.5 mM tBHP was incubated with a 20 µM concentration of the fluorescent dye CM-H_2_DCFDA. CM-H_2_DCFDA is oxidized by superoxide/ROS into the fluorescent dye DCF, which was analyzed by fluorescence microscopy and quantified with ImageJ. The scale bar indicates 0.2 mm. Statistical analysis of the lifespan was performed using GraphPad 9 and *p* values were calculated by the log-rank test. Numerical data were analyzed by Student’s t-test and values were presented as mean ± SD. Statistical significance is indicated as *p < 0.05, **p < 0.01, and ***p < 0.001, compared to the Ctrl group.

To systematically assess stress resistance, we exposed 100, 200, and 400 μM 20(S)-PPD-treated N2 worms to heat, oxidative, and chemical stressors (paraquat [PQ]). A concentration of 9.125 mM tBHP was utilized to induce oxidative stress (Li et al., 2019). It was found that 20(S)-PPD treatment markedly improved oxidative stress tolerance in N2 worms, with the survival rate extensions of 6.315, 17.004, and 13.172% at concentrations of 100, 200, and 400 μM, respectively (Figure 3A). To induce heat stress, 20(S)-PPD-treated N2 worms were kept in an incubator at 35 °C until death. It was found that all doses of 20(S)-PPD dose-dependently extended the mean lifespan of the worms relative to the controls (Figure 3B). Adult worms were also exposed to PQ, showing that 200 and 400 μM 20(S)-PPD enhanced resistance, although concentrations of 100 μM were ineffective (Figure 3C). This, in agreement with the lifespan assessment results, 20(S)-PPD treatment could protect against various forms of stress in *C. elegans*.

It is known that increased ROS resulting from mitochondrial dysfunction can damage cellular structures and contribute to age-related deterioration (Davalli et al., 2016). DHE which forms 2-hydroxyethidium (2-EOH) after superoxide oxidation and can be taken up by the worms was used to evaluate ROS levels in *C. elegans* after exposure to 20(S)-PPD at day 10. Evaluation of fluorescence intensities indicated that all doses of 20(S)-PPD treatment markedly reduced the levels of ROS induced by aging, with 200 μM 20(S)-PPD showing optimal inhibition (Figure 3D). In addition, ROS induced by 5.5 mM tBHP was measured by incubating cell lysates with the fluorescence probe CM-H_2_DCFDA, as previously described. The acetate groups of CM-H_2_DCFDA undergo cleavage by intracellular esterases while its thiol-reactive chloromethyl group reacts with intracellular glutathione and other thiols. Subsequent oxidation generates a fluorescent adduct, which can be measured to determine ROS levels. As illustrated in Figure 3E, 200 and 400 μM 20(S)-PPD markedly reduced ROS levels induced by 5.5 mM tBHP, with declines in the fluorescence intensities of 20.22% and 24.56% respectively, relative to the untreated group. Taken together, these results indicated that 20(S)-PPD enhances resistance to multiple forms of stress and reduces ROS levels induced by both aging and oxidative stress. In addition, based on the results of lifespan and aging-associated phenotypes, 200 μM 20(S)-PPD appears to be the optimal pharmacodynamic dose.

### 3.3 20(S)-PPD binds to the IR *in silico* and *in vitro*

**FIGURE 4:**
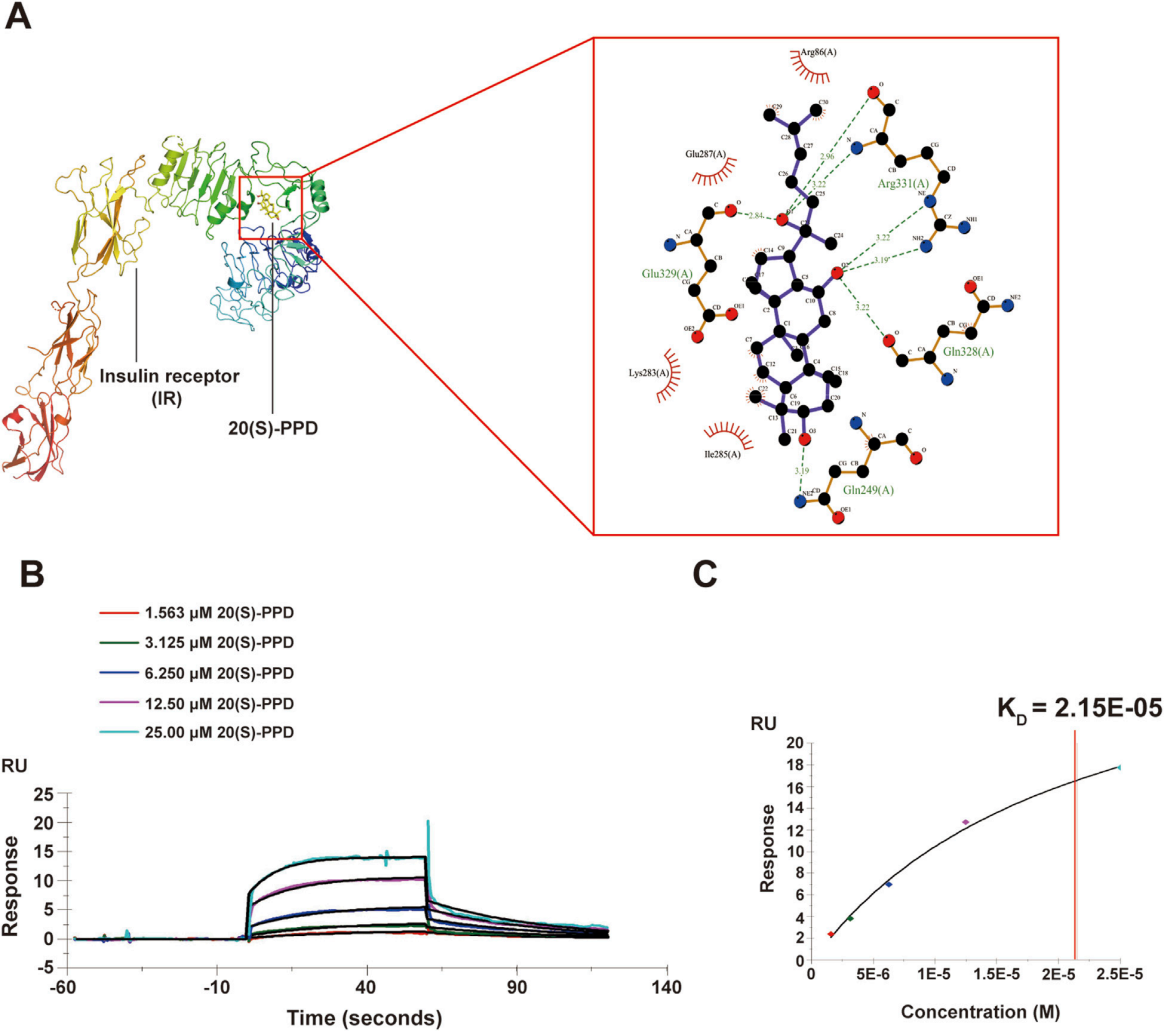
20(S)-PPD bounds to IR *in vitro*. **(A)** 3D diagrams (left) and 2D (right) diagrams of the docked structure of 20(S)-PPD in the active domain of the IR. **(B,C)** SPR analysis of 20(S)-PPD binding to IR protein **(B)** and K_D_ value was calculated **(C)**.

To elucidate the mechanisms by which the 20(S)-PPD extended lifespan, SwissTargetPrediction was utilized for predicting potential 20(S)-PPD targets. The canonical SMILES of 20(S)-PPD were obtained from the PubChem database and loaded into SwissTargetPrediction for target prediction. One hundred proteins known to be involved in aging were screened as potential targets, using a condition where “Probability”> 0. This identified 57 proteins as candidate targets of 20(S)-PPD-induced anti-aging effects (Supplementary Table S4). The IR is known to be linked with increased lifespan and is evolutionarily conserved (Kimura et al., 1997). The molecular docking results showed that the binding energy of 20(S)-PPD to IR was −7.4 kcal/mol, and 20(S)-PPD interacted with IR mainly through hydrogen bonds (involving Gln249, Gln328, Arg331, and Glu329) and hydrophobic bonds (involving Arg86, Glu287, Lys283, and Ile285). These results indicate a potential interaction between 20(S)-PPD and IR, as depicted in Figure 4A. To further verify this interaction, we assessed the affinity of human IR proteins for 20(S)-PPD using SPR. As shown in Figures 4B,C, 20(S)-PPD dose-dependently bound to IR with a K_D_ (equilibrium dissociation constant) value of 8.59 μM. These results indicated that 20 (S)-PPD has ability to bind to IR *in vitro* and it was speculated that IR is required for PPD-induced longevity.

### 3.4 20(S)-PPD extends lifespan via DAF-2/insulin/IGF-1R signaling (IIS) pathway in *Caenorhabditis elegans*

**FIGURE 5:**
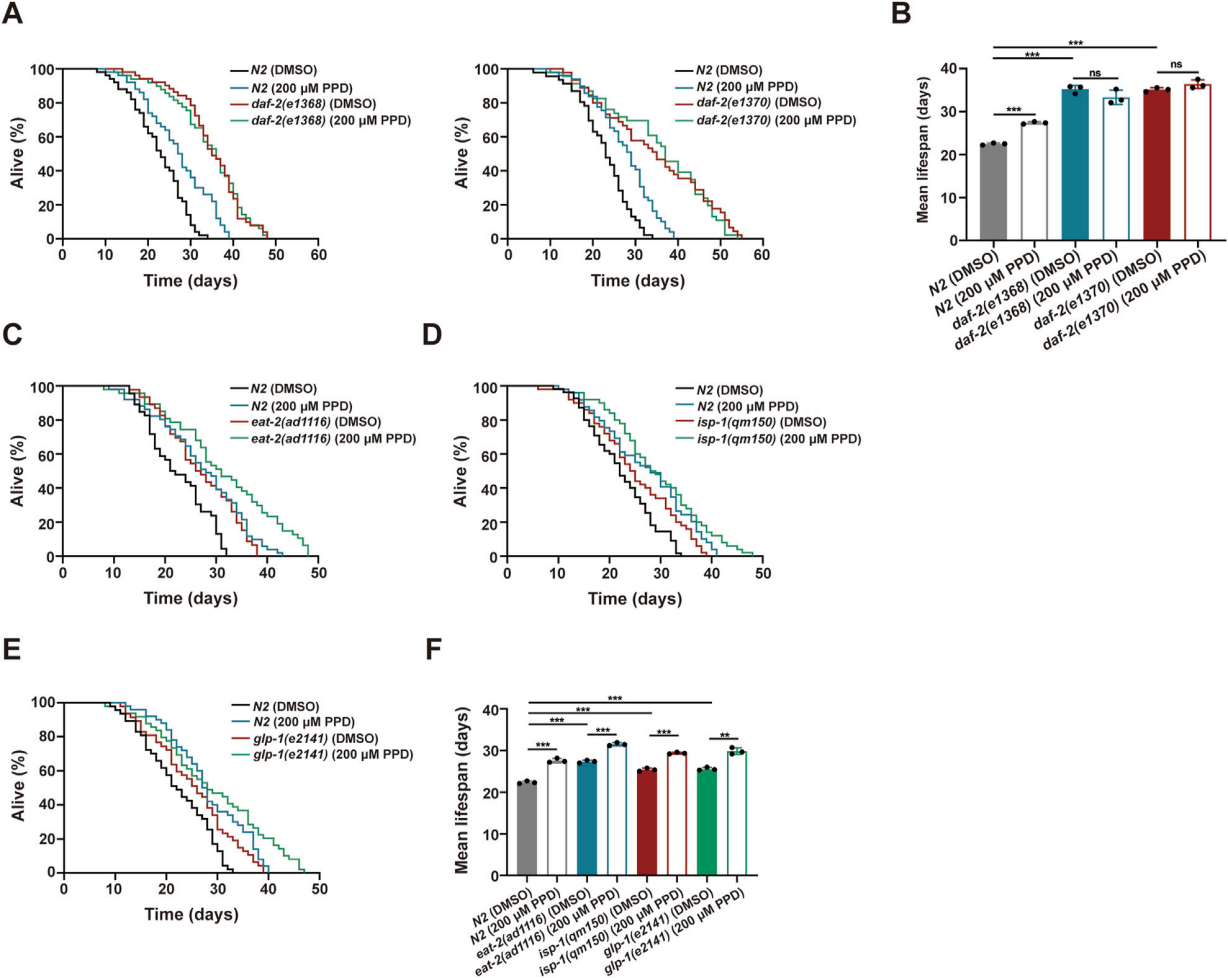
20(S)-PPD increases (C) *elegans* lifespan via IR/DAF-2 signaling pathway. The strains utilized included N2, wild type; CB1370, *daf-2(e1370);* CB1368, *daf-2(e1368)*; MQ887, *isp-1(qm150)*; DA1116, *eat-2(ad1116);* CB4037, *glp-1(e2141)*. Ctrl, treated with DMSO. PPD, treated with 200 μM 20(S)-PPD. **(A,B)** Lifespan experiments were performed on *daf-2* gene mutants, including *daf-2(e1368)* and *daf-2(e1370),* exposed to 200 μM 20(S)-PPD. **(A)** The survival curve and **(B)** the average lifespan of each group (n = 3). For each group, >30 worms were counted as detailed in the methods. **(C–F)** Lifespan assays for mutants of longevity-related pathway genes after 200 μM 20(S)-PPD treatment. **(C–E)** The survival curve of mutants of longevity-related pathway genes including *eat-2(ad1116)* **(C)**, *isp-1(qm150)* **(D)**, and *glp-1(e2141)* **(E)** exposed to 200 μM 20(S)-PPD. **(F)** The average lifespan of each group (n = 3). For each group, >30 worms were counted as detailed in the methods. Statistical analysis of the lifespan was performed using GraphPad 9 and *p* values were calculated by the log-rank test. Numerical data were analyzed by Student’s t-test and values were presented as mean ± SD. ns, no significance; statistical significance is indicated as **p < 0.01 and ***p < 0.001.

To confirm the above hypothesis, two mutants of *daf-2*, the only IR homolog in *C. elegans*, *daf-2(e1368)* and *daf-2(e1370),* were used assessing lifespan (Murphy and Hu, 2013). The survival curves and mean lifespan measurements showed that 200 μM 20(S)-PPD did not prolong the lifespan of the mutants (Figures 5A,B). We then investigated other pathways potentially influencing lifespan in *C. elegans*, including dietary restriction (Cao et al., 2023), germline signaling, and mitochondrial respiration (Maselli and Camilleri, 2021; Yang and Hekimi, 2010). Treatment with 20(S)-PPD markedly increased the mean lifespan of the *eat-2(ad1116)* mutant, a nicotinic acetylcholine receptor mutant with a long life (Cao et al., 2023). This mutant imitates food restriction by suppressing pharyngeal pumping and food intake (Figures 5C,F). Similarly, 20(S)-PPD treatment further significantly increased the lifespan of both the long-lived mitochondrial complex III mutant *isp-1(qm150)* and the germline deletion mutant *glp-1(e2141)* (Maselli and Camilleri, 2021; Yang and Hekimi, 2010) (Figures 5D–F). These results indicate that apart from the *daf-2* mutant, 20(S)-PPD effectively prolonged survival in all mutants, demonstrating that IIS pathway was linked to the life-extending effects of 20(S)-PPD.

### 3.5 20(S)-PPD-mediated longevity and enhancement of stress tolerance depends on DAF-16/FOXO

**FIGURE 6:**
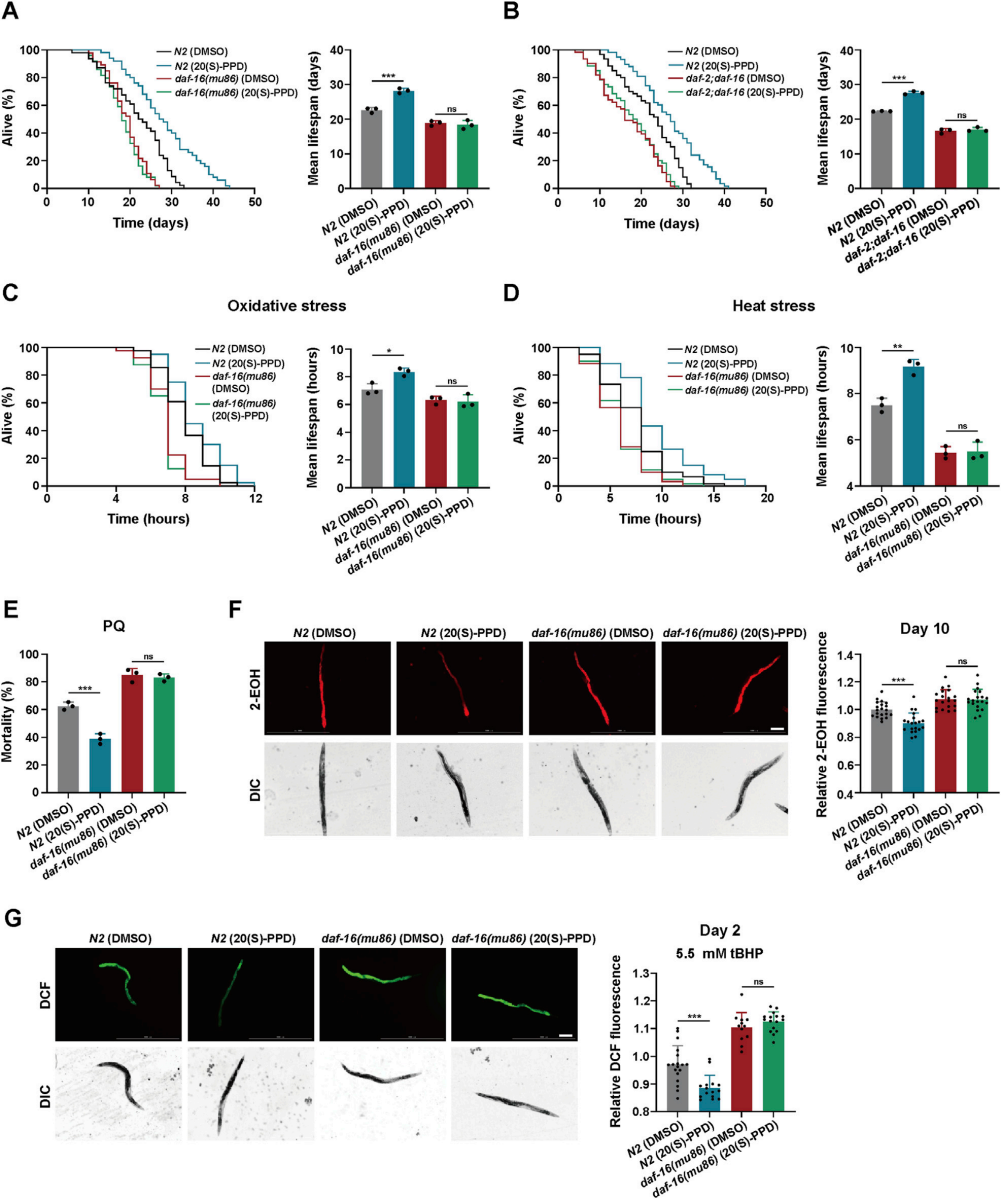
20(S)-PPD increases (C) *elegans* lifespan and stress tolerance via FOXO/DAF-16. The strains utilized included N2, wild type; CF1308, *daf-16(mu86)*; *daf-2;daf-16*, *daf-2(e1370);daf-16(mu86).* DMSO, treated with DMSO. 20(S)-PPD, treated with 200 μM 20(S)-PPD. **(A,B)** Survival plot and average lifespan for *daf-16* gene mutants **(A)** and *daf-2;daf-16* double mutants **(B)** exposed to 200 μM 20(S)-PPD. For each group, more than 30 worms were counted as detailed in the methods (n = 3). **(C,D)** Survival plot and average lifespan for *daf-16* gene mutants exposed to 200 μM 20(S)-PPD. **(C)** Oxidative stress. **(D)** Heat stress. For each group, more than 30 worms were counted as detailed in the methods (n = 3). **(E)** The resistance to oxidative stress of *daf-16* gene mutants to 200 mM PQ (n = 50 worms for each group each time, experiment repeated 3 times). **(F)** ROS levels of 20 adult *daf-16* gene mutants exposed to 200 μM 20(S)-PPD at day 10 using DHE staining. The scale bar indicates 0.2 mm. **(G)** ROS levels of tBHP-treated 20 adult *daf-16* gene mutants exposed to 200 μM 20(S)-PPD at day 2 using CM-H_2_DCFDA staining. The scale bar indicates 0.2 mm. The fluorescence intensity was analyzed with ImageJ. Statistical analysis of the lifespan was performed using GraphPad 9 and *p* values were calculated by the log-rank test. Numerical data were analyzed by Student’s t-test and values were presented as mean ± SD. ns, no significance; statistical significance is indicated as *p < 0.05, **p < 0.01, and ***p < 0.001.

DAF-16, the only FOXO3A homolog in *C. elegans*, is a well-documented transcription factor that regulates longevity through insulin signaling (Su et al., 2018). For further investigation, the *daf-16(mu86)* strain comprising a mutant *daf-16* form was utilized to identify survival in the presence or absence of 20(S)-PPD. It was found that 200 μM 20(S)-PPD had no significant effect on the lifespan of *daf-16(mu86)*, suggesting that DAF-16/FOXO is crucial for 20(S)-PPD-induced longevity (Figure 6A). Previous studies have indicated the involvement of DAF-16 in several aging-related pathways besides the insulin pathway. To clarify whether DAF-16 mediated 20(S)-PPD-induced longevity depends on the IIS pathway, we exposed *daf-2(e1370)*;*daf-16(mu86)* double-mutant worms to 200 μM 20(S)-PPD and assessed their lifespan. It was observed that 200 μM 20(S)-PPD did not prolong the lives of *daf-2(e1370)*;*daf-16(mu86)* double-mutant worms (Figure 6B), demonstrating that 20(S)-PPD mediated longevity via the DAF-2/DAF-16 insulin axis.

To determine whether DAF-16/FOXO is required for 20(S)-PPD-mediated enhancement of stress tolerance, the tolerance of the *daf-16(mu86)* strain to heat, oxidative stress, and chemical stress after exposure to 200 μM 20(S)-PPD was assessed. This showed that 200 μM 20(S)-PPD offered no significant protection against these stressors, in contrast with the effects observed in N2 worms (Figures 6C–E). Thus, 20(S)-PPD-mediated inhibition of ROS accumulation triggered by aging and oxidative stress depends on DAF-16/FOXO (Figures 6F,G). These findings suggest that 20(S)-PPD mediated longevity, enhanced stress tolerance, and ROS reduction via DAF-16/FOXO.

### 3.6 20(S)-PPD activates DAF-16/FOXO to promote expression of antioxidant and detoxification-associated genes

**FIGURE 7:**
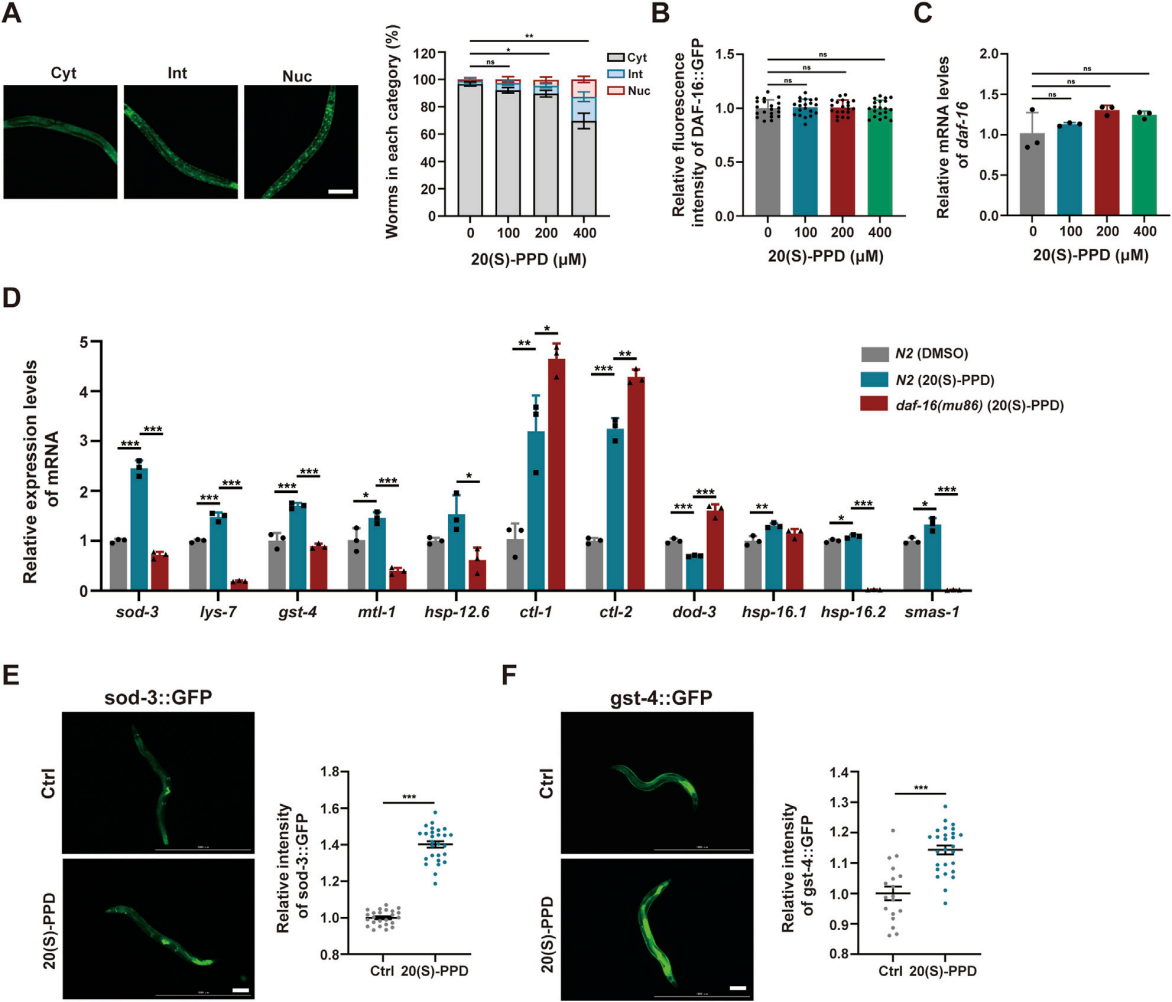
The nuclear localization of FOXO/DAF-16 mediated by 20(S)-PPD to modulate its target genes expression. The strains utilized included N2, wild type; TJ356, *zIs356[daf-16::gfp]*; CF1553, *muIs84 [(pAD76) sod-3p::GFP + rol-6(su1006)]*; CL2166, *dvIs19[(pAF15) gst-4p::GFP::NLS]*; CF1308, *daf-16(mu86).* Ctrl, treated with DMSO. 20(S)-PPD, treated with 100, 200, or 400 μM 20(S)-PPD. **(A)** Representative images of specific cellular localization patterns of DAF-16:GFP in the intermediate, cytoplasmic, and nuclear compartments (left), and the statistical data of DAF-16:GFP cellular localization patterns after 20(S)-PPD treatment (right). **(B)** Corresponding quantification of fluorescence intensity values of DAF-16:GFP. **(C)** RT-qPCR was employed to measure the mRNA levels of *daf-16* genes after 20(S)-PPD treatment. **(D)** RT-qPCR was carried out to assess the mRNA levels of 11 *daf-16* target genes in both N2 and *daf-16* mutant strains after 20(S)-PPD treatment. **(E,F)** The post-20(S)-PPD treatment green fluorescence of CF1553 worms with a SOD-3:GFP fusion gene **(E)** and CL2166 worms with a GST-4:GFP fusion gene **(F)** were detected by fluorescence microscopy. Representative images are shown on the left, and ImageJ was employed to assess the relative fluorescence intensity (right). Scale bars: 200 µm. Statistical analysis were analyzed by Student’s t-test and values were presented as mean ± SD. Statistical significance is indicated as *p < 0.05, **p < 0.01, and ***p < 0.001.

Activation of DAF-16 by dephosphorylation leads to its migration to the nucleus where it enhances the transcription of antioxidant and detoxification-related genes, thereby increasing the lifespan of *C. elegans* (Sun et al., 2017). To elucidate whether 20(S)-PPD modulates DAF-16-promoted transcription, transgenic worms TJ356 (DAF-16:GFP) expressing a functional DAF-16GFP fusion protein were utilized. The TJ356 strains were classified into nuclear, intermediate, and cytoplasmic groups to analyze and score the nuclear localization of DAF-16 in the gut. The results indicated that 200 and 400 μM 20(S)-PPD treatment markedly elevated nuclear accumulation of DAF-16:GFP (Figure 7A). Moreover, *daf-16* mRNA and protein levels were unaffected at all doses of 20(S)-PPD (Figures 7B,C). The findings suggested that 20(S)-PPD promoted the nuclear localization of DAF-16/FOXO.

Due to the reported antioxidant and detoxification effects of DAF-16/FOXO target genes, the mRNA levels of *sod-3*, *lys-7*, *gst-4*, *mtl-1*, *hsp-12.6*, *ctl-1*, *ctl-2*, *dod-3*, *hsp-16.1*, *hsp-16.2*, and *sms-1* were assessed via RT-qPCR. Consistent with earlier results, the mRNA levels of 10 of 11 *daf-16* target genes were markedly altered in the *daf-16* mutants relative to the N2 worms (Figure 7D). The mRNA expression of *lys-7*, *mtl-1*, *hsp-12.6*, *dod-3*, *sod-3*, *hsp-16.2*, *gst-4* and *sms-1* were rescued after 20(S)-PPD treatment (Figure 6D). The SOD-3 and GST-4 protein levels were evaluated via the GFP reporter strains CL2166 and CF1553 harboring the *gst-4*-GFP and *sod-3*-GFP fusion genes, respectively (Balkrishna et al., 2023). Microscopic analysis of GFP fluorescence demonstrated notably enhanced levels of these proteins after 200 μM 20(S)-PPD treatment (Figures 7E,F). These results indicate that 20(S)-PPD induces the migration of FOXO/DAF-16 to the nucleus, which then interacts with the promoter regions of genes such as *sod-3* and *gst-4* to induce their transcription, leading to lifespan extension and stress resistance.

## 4 Discussion

**FIGURE 8:**
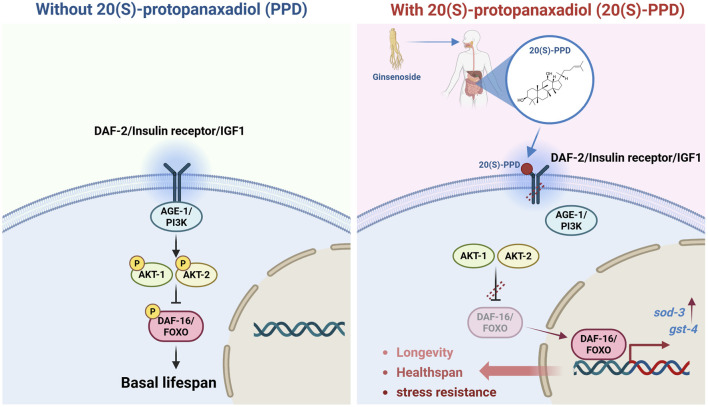
Schematic illustration of the regulatory effects and mechanism of 20(S)-PPD on lifespan, healthspan, and stress resistance via insulin/IGF-1 signaling pathway, detailed in the results section.

Ginseng has been used for 5,000 years in traditional Chinese medicine due to its various healing and beneficial properties. Ginsenosides, a group of triterpene glycosides, are the active chemicals found in ginseng (Chopra et al., 2023). Chemically, these glycosides may be categorized into two groups: 20(S)-protopanaxatriol (20(S)-PPT) and 20(S)-protopanaxadiol (20(S)-PPD) (Mancuso and Santangelo, 2017). Of these, 20(S)-PPD contains the most ginsenosides, including ginsenoside Rb1, Rg3, Rb2, Rc, Rd, Rb3, and Rh2 (Jo et al., 2021). Previous research has shown that 20(S)-PPD has various beneficial properties, protecting against fatigue, inflammation, and cancer (Lu et al., 2018). Research on epithelial cells from elderly individuals (over 65 years old) revealed that 20(S)-PPD could significantly enhance the production of type-I collagen, fibrillin-1, and elastin, indicating likely anti-aging benefits (Lee et al., 2021). However, the efficacy of 20(S)-PPD in prolonging lifespan and anti-aging requires *in vivo* evidence. In this study, *C. elegans* was employed as an animal model to elucidate the modulatory effects of 20(S)-PPD on aging, and further elucidate the mechanism. The results indicated that 20(S)-PPD could extend lifespan and healthspan, and enhance resistance to stress by activation of the DAF-2/insulin/IR axis and the transcription factor DAF-16/FOXO (Figure 8).

IR and IGF1R belong to the receptor tyrosine kinase (RTK) subfamily and have crucial physiological roles (Cai et al., 2022). Dysregulation of IR signaling can lead to human diseases like diabetes, aging, and cancer (Kenyon, 2011). The IR receptors form a stable dimers linked by disulfide bonds and inter-protomer contacts, and cannot bind ligands, unlike other monomeric RTKs in the unliganded state (Hubbard, 2013). IGF1 induces structural alterations and stabilizes the active IGF1R and IR conformations, enabling signaling cascade activation (Uchikawa et al., 2019). The c-terminal region of IR has several tyrosine phosphorylation sites that facilitate its interaction with proteins containing Src-homology domain 2 (SH2), such as PI3K and protein tyrosine phosphatases (Chakraborty et al., 2013). The N-terminal regions of IR family proteins are strongly conserved and contain two domains involved in receptor recruitment: the phosphotyrosine binding (PTB) and pleckstrin homology (PH) domains (Hubbard, 2013). The PTB domain contains a tyrosine residue that recognizes the asparagine-proline-glutamate-phosphotyrosine sequence in activated receptors (Boucher et al., 2014). The use of the “SwissTargetPrediction” algorithm and molecular docking indicated that 20(S)-PPD could interact with IGF-1. This binding was facilitated by the formation of hydrogen bonds involving Gln249, Gln328, Arg331, and Glu329, as well as hydrophobic interactions with Arg86, Glu287, Lys283, and Ile285. Furthermore, these interactions were predominantly concentrated within the PTB domain. In addition, SPR was utilized to clarify the interaction between IR and 20(S)-PPD. However, further confirmation is needed to determine whether 20(S)-PPD interacts directly with IR to control receptor recruitment. Moreover, lifespan assays of the *daf-2* mutant indicated that 20(S)-PPD prolonged *C. elegans* longevity via DAF-2 insulin/IR. However, the specific mechanism by which 20(S)-PPD interacts with DAF-2 to induce the life-extending effects of 20(S)-PPD remains unclear and requires more research.

Over the past three decades, significant progress has revealed that conserved genetic pathways modulate animal longevity. The first identified and extensively studied gene in this context is *daf-2*, a single-gene mutation in the insulin/IGF-1 signaling (IIS) pathway, which doubles the lifespan of *C. elegans* (Zhang et al., 2022). Beyond an extended lifespan, *daf-2* mutants show a wide range of pleiotropic effects, including developmental abnormalities, reduced brood size, and increased fat accumulation (Gems et al., 1998; Kimura et al., 1997). Similar phenotypes have also been observed in *daf-2* mouse mutants (Bartke, 2008). Our findings suggest that while both *daf-2* mutation and 20(S)-PPD treatment extend longevity, they have contrasting effects on healthspan: 20(S)-PPD improves mobility and does not compromise reproductive capacity in *C. elegans*. Tissue-specific studies have shown that intracellular DAF-2-to-DAF-16 signaling in the intestine is a major regulator of lifespan, whereas signaling in hypodermis, neurons, and germline contributes less significantly (Zhang et al., 2022). Intestine-specific loss of *daf-2* activates *daf-16* both within and outside the intestine, produces minimal developmental and reproductive defects, and extends lifespan by 94%, partially dependent on non-intestinal *daf-16* (Zhang et al., 2022). Based on these findings and the *in vivo* metabolism of 20(S)-PPD, we speculate that 20(S)-PPD may act as a partial inhibitor of *daf-2*, exerting its effects on lifespan and healthspan by specifically targeting intestine-expressed *daf-2*. However, this hypothesis requires further validation through *in vivo* and *in vitro* experiments, as well as pharmacokinetic studies in aging mice.

It has been found that 20(S)-PPD can regulate multiple signaling axes. Studies conducted on A549 cells have shown that 20(S)-PPD can interfere with the EGFR/MAPK axis by binding directly to EGFR (Zhang et al., 2020). Phosphorylation and accumulation of Raf-1, BRAF, MEK, EGFR, Ras, and ERK were found to be altered after 20(S)-PPD treatment, leading to cell cycle arrest (Zhang et al., 2020). 20(S)-PPD was found to prevent the release of inflammatory cytokines and the formation of osteoclasts by suppressing the MAPK and NF-κB pathways (Pan et al., 2018), which is consistent with a report that 20(S)-PPD can enhance worm motility. An *in vitro* study showed that human umbilical vein endothelial cells (HUVECs) can undergo apoptosis under the action of 20(S)-PPD, through activation of the PERK-eIF2alpha-ATF4 axis (Wang et al., 2019). Moreover, both cellular and animal studies showed that 20(S)-PPD treatment was able to modulate the AMPK/STING axis (Ren et al., 2023). Thus, more research is required to investigate whether 20(S)-PPD-induced extension of healthspan occurs through pathways regulated by 20(S)-PPD or through other pathways.

Lipofuscin, also termed age pigment, is an intralysosomal polymeric material that lysosomal hydrolases cannot degrade nor exocytose (Terman and Brunk, 2004). It is considered a biomarker of aging, with accumulation inversely related to longevity (Yang and Hekimi, 2010). Furthermore, there is compelling evidence suggesting that the accumulation of lipofuscin over time reduces the ability of cells to adapt and contributes to the onset of age-associated disorders such as heart disease, neurological disorders, and macular degeneration (Jolly et al., 1995; Nakano et al., 1995). However, the utility of lipofuscin as a universal and reliable marker faces significant limitations, primarily its high variability and heterogeneity. Firstly, its accumulation rates and levels are strongly influenced by genetic background, lifestyle factors (e.g., diet and stress), and environmental exposures (e.g., toxins and UV light), leading to substantial inter-individual differences (Baldensperger et al., 2024; de Gritz et al., 1994). Secondly, there is pronounced tissue and cell-type specificity; lipofuscin’s accumulation is fastest in post-mitotic cells, and its composition and morphology can vary even between different cells within the same tissue (Crouch et al., 2015). Thirdly, its relationship with chronological age is not strictly linear, and it is highly sensitive to pathological states, such as neurodegenerative diseases, lysosomal storage disorders, and vitamin deficiencies, making it impossible to distinguish aging from disease interference (Yang et al., 2025). Finally, its causal role remains ambiguous (is it a passive byproduct or an active driver of aging?), and universal healthy thresholds are lacking (Szweda et al., 2003). Therefore, interpreting lipofuscin data requires extreme caution, considering its complex context-dependence. It needs to be combined with other biomarkers into a composite assessment system, rather than serving as a single, universal indicator of aging rate or biological age.

Here, it was found that intestinal lipofuscin levels were markedly higher on day 11 than on day 7, while accumulation was suppressed after 20(S)-PPD treatment. The production of lipofuscin is induced by ROS accumulation via triggering peroxidation of auto-/heterophagocytosed macromolecules (Boulton et al., 2004). However, it remains uncertain whether the 20(S)-PPD inhibition of lipofuscin accumulation associated with aging is mediated by ROS. Further *in vivo* investigation is required to construct a model of oxidative injury in nematodes.

Previous studies have indicated that neurodegenerative diseases such as Alzheimer’s disease (AD) and Parkinson’s disease (PD) are primarily age-related and typically present with cognitive impairments (Hou et al., 2019). Moreover, as aging and neurodegeneration progress, a substantial proportion of individuals show noticeable cognitive decline (Bellelli et al., 2025). Therefore, we evaluated the neuroprotective effects of 20(S)-PPD in age-related neurodegeneration and cognitive degeneration. An aversive olfactory learning assay was carried out to evaluate the regulatory effects of 20(S)-PPD on aging-related cognitive degradation (Rozanov et al., 2020). The chemotaxis index (CI) is negatively associated with cognitive ability in worms. It was found that compared with young worms, the CI of old worms increased markedly indicating reduced cognitive ability, while 20(S)-PPD could dose-dependently rescue cognitive decline in old individuals (Supplementary Figure S1A). The AM44 strain expressing polyglutamine (PolyQ) proteins in the worm nervous system is used as a model of polyQ-associated neurodegeneration in *C. elegans* (Yang et al., 2024). The results indicated that 20(S)-PPD was effective in preventing paralysis in AM44 worms, with reduction rates of 20.804, 41.540, and 24.839% at concentrations of 100, 200, and 400 μM, respectively (Supplementary Figure S1B). These evidences are preliminary exploration of 20(S)-PPD’s neuroprotective effects against aging-associated neurodegeneration. Further *in vivo* investigation is required to clarify the exact neuroprotective efficacy and mechanism of 20(S)-PPD.

## Data Availability

The raw data supporting the conclusions of this article will be made available by the authors, without undue reservation.
