# Supplementary material for: 20(S)-protopanaxadiol prolongs lifespan and enhances stress resistance in Caenorhabditis elegans via the insulin/IGF-1 signaling pathway
Source: Front Pharmacol. 2025 Oct 14;16:1657436. doi: 10.3389/fphar.2025.1657436 (PMC12558884; doi:10.3389/fphar.2025.1657436)
Supplement: Supplementary file 1 [file Table4.doc]

**Table S4. The candidate targets of 20(S)-PPD-induced anti-aging predicted by SwissTargetPrediction**

| **Protein** | **Common name** | **Uniprot ID** | **ChEMBL ID** | **Target Class** | **Probability** |
| --- | --- | --- | --- | --- | --- |
| Protein-tyrosine phosphatase 1B | PTPN1 | P18031 | CHEMBL335 | Phosphatase | 0.134155403 |
| Androgen Receptor | AR | P10275 | CHEMBL1871 | Nuclear receptor | 0.109013468 |
| Cytochrome P450 2C19 | CYP2C19 | P33261 | CHEMBL3622 | Cytochrome P450 | 0.109013468 |
| Muscarinic acetylcholine receptor M2 | CHRM2 | P08172 | CHEMBL211 | Family A G protein-coupled receptor | 0.100634432 |
| Norepinephrine transporter | SLC6A2 | P23975 | CHEMBL222 | Electrochemical transporter | 0.100634432 |
| Serotonin transporter | SLC6A4 | P31645 | CHEMBL228 | Electrochemical transporter | 0.100634432 |
| LXR-alpha | NR1H3 | Q13133 | CHEMBL2808 | Nuclear receptor | 0.100634432 |
| Cytochrome P450 19A1 | CYP19A1 | P11511 | CHEMBL1978 | Cytochrome P450 | 0.100634432 |
| Acetylcholinesterase | ACHE | P22303 | CHEMBL220 | Hydrolase | 0.100634432 |
| Estrogen receptor alpha | ESR1 | P03372 | CHEMBL206 | Nuclear receptor | 0.100634432 |
| 11-beta-hydroxysteroid dehydrogenase 1 | HSD11B1 | P28845 | CHEMBL4235 | Enzyme | 0.100634432 |
| Cytochrome P450 51 | CYP51A1 | Q16850 | CHEMBL3849 | Cytochrome P450 | 0.100634432 |
| HMG-CoA reductase | HMGCR | P04035 | CHEMBL402 | Oxidoreductase | 0.100634432 |
| Potassium-transporting ATPase alpha chain 2 | ATP12A | P54707 | CHEMBL2933 | Primary active transporter | 0.100634432 |
| Nuclear receptor ROR-gamma | RORC | P51449 | CHEMBL1741186 | Nuclear receptor | 0.100634432 |
| Phosphodiesterase 10A | PDE10A | Q9Y233 | CHEMBL4409 | Phosphodiesterase | 0.100634432 |
| Butyrylcholinesterase | BCHE | P06276 | CHEMBL1914 | Hydrolase | 0.100634432 |
| Nuclear receptor subfamily 1 group I member 3 | NR1I3 | Q14994 | CHEMBL5503 | Nuclear receptor | 0.100634432 |
| UDP-glucuronosyltransferase 2B7 | UGT2B7 | P16662 | CHEMBL4370 | Enzyme | 0.100634432 |
| Niemann-Pick C1-like protein 1 | NPC1L1 | Q9UHC9 | CHEMBL2027 | Other membrane protein | 0.100634432 |
| Carboxylesterase 2 | CES2 | O00748 | CHEMBL3180 | Enzyme | 0.100634432 |
| Vitamin D receptor | VDR | P11473 | CHEMBL1977 | Nuclear receptor | 0.100634432 |
| Subtilisin/kexin type 7 | PCSK7 | Q16549 | CHEMBL2232 | Protease | 0.100634432 |
| Cyclooxygenase-2 | PTGS2 | P35354 | CHEMBL230 | Oxidoreductase | 0.100634432 |
| ALK tyrosine kinase receptor | ALK | Q9UM73 | CHEMBL4247 | Kinase | 0.100634432 |
| Cytochrome P450 2C9 | CYP2C9 | P11712 | CHEMBL3397 | Cytochrome P450 | 0.100634432 |
| Cytochrome P450 3A4 | CYP3A4 | P08684 | CHEMBL340 | Cytochrome P450 | 0.100634432 |
| Proteinase-activated receptor 1 | F2R | P25116 | CHEMBL3974 | Family A G protein-coupled receptor | 0.100634432 |
| PI3-kinase p110-delta subunit | PIK3CD | O00329 | CHEMBL3130 | Enzyme | 0.100634432 |
| PI3-kinase p110-beta subunit | PIK3CB | P42338 | CHEMBL3145 | Enzyme | 0.100634432 |
| PI3-kinase p110-gamma subunit | PIK3CG | P48736 | CHEMBL3267 | Enzyme | 0.100634432 |
| PI3-kinase p110-alpha subunit | PIK3CA | P42336 | CHEMBL4005 | Enzyme | 0.100634432 |
| Testis-specific androgen-binding protein | SHBG | P04278 | CHEMBL3305 | Secreted protein | 0.100634432 |
| Fatty acid synthase | FASN | P49327 | CHEMBL4158 | Transferase | 0.100634432 |
| Vasopressin V1a receptor | AVPR1A | P37288 | CHEMBL1889 | Family A G protein-coupled receptor | 0.100634432 |
| Melatonin receptor 1A | MTNR1A | P48039 | CHEMBL1945 | Family A G protein-coupled receptor | 0.100634432 |
| Melatonin receptor 1B | MTNR1B | P49286 | CHEMBL1946 | Family A G protein-coupled receptor | 0.100634432 |
| Tyrosine-protein kinase BRK | PTK6 | Q13882 | CHEMBL4601 | Kinase | 0.100634432 |
| Estrogen-related receptor alpha | ESRRA | P11474 | CHEMBL3429 | Nuclear receptor | 0.100634432 |
| Estrogen-related receptor beta | ESRRB | O95718 | CHEMBL3751 | Nuclear receptor | 0.100634432 |
| Insulin receptor | INSR | P06213 | CHEMBL1981 | Kinase | 0.100634432 |
| Phosphodiesterase 2A | PDE2A | O00408 | CHEMBL2652 | Phosphodiesterase | 0.100634432 |
| Phosphodiesterase 4B | PDE4B | Q07343 | CHEMBL275 | Phosphodiesterase | 0.100634432 |
| Collagen type IV alpha-3-binding protein | COL4A3BP | Q9Y5P4 | CHEMBL3399913 | Unclassified protein | 0.100634432 |
| Cytochrome P450 17A1 | CYP17A1 | P05093 | CHEMBL3522 | Cytochrome P450 | 0.100634432 |
| Adenosine A1 receptor | ADORA1 | P30542 | CHEMBL226 | Family A G protein-coupled receptor | 0.100634432 |
| Calpain 2 | CAPN2 | P17655 | CHEMBL2382 | Protease | 0.100634432 |
| Adenosine A2a receptor | ADORA2A | P29274 | CHEMBL251 | Family A G protein-coupled receptor | 0.100634432 |
| MAP kinase p38 alpha | MAPK14 | Q16539 | CHEMBL260 | Kinase | 0.100634432 |
| Cyclooxygenase-1 | PTGS1 | P23219 | CHEMBL221 | Oxidoreductase | 0.100634432 |
| Liver glycogen phosphorylase | PYGL | P06737 | CHEMBL2568 | Enzyme | 0.100634432 |
| c-Jun N-terminal kinase 1 | MAPK8 | P45983 | CHEMBL2276 | Kinase | 0.100634432 |
| Interleukin-6 receptor subunit beta | IL6ST | P40189 | CHEMBL3124734 | Membrane receptor | 0.100634432 |
| Cytochrome P450 24A1 | CYP24A1 | Q07973 | CHEMBL4521 | Enzyme | 0.100634432 |
| Vascular endothelial growth factor receptor 2 | KDR | P35968 | CHEMBL279 | Kinase | 0.100634432 |
| p53-binding protein Mdm-2 | MDM2 | Q00987 | CHEMBL5023 | Other nuclear protein | 0.100634432 |
| Smoothened homolog | SMO | Q99835 | CHEMBL5971 | Frizzled family G protein-coupled receptor | 0.100634432 |
