# Supplementary material for: 20(S)-protopanaxadiol prolongs lifespan and enhances stress resistance in Caenorhabditis elegans via the insulin/IGF-1 signaling pathway
Source: Front Pharmacol. 2025 Oct 14;16:1657436. doi: 10.3389/fphar.2025.1657436 (PMC12558884; doi:10.3389/fphar.2025.1657436)
Supplement: Supplementary file 2 [file Table3.doc]

**Table S3. The primer sequences using for RT-qPCR assays**

| **Primers** | **Sequences of primers of mRNA** |
| --- | --- |
| *act-1* forward | 5′-CTACGAACTTCCTGACGGACAAG-3′ |
| *act-1* reverse | 5′-CCGGCGGACTCCATACC-3′ |
| *sod-3* forward | 5′-TGGCTAAGGATGGTGGAGAA-3′ |
| *sod-3 reverse* | 5′-GCCTTGAACCGCAATAGTGAT-3′ |
| *mtl-1* forward | 5′-ATGGCTTGCAAGTGTGACTGCAAAAACAAGC-3′ |
| *mtl-1* reverse | 5′-TTAATGAGCCGCAGCAGTTCCCTGGTGTTGATGGG-3′ |
| *smas-1* forward | 5′-CGTTGGGTAGCCACCTCATT-3′ |
| *smas-1* reverse | 5′-TCTCGGCGGTCTTCTCGTAG-3′ |
| *hsp-12.6* forward | 5′-ATGATGAGCGTTCCAGTGATGGCTGACG-3′ |
| *hsp-12.6* reverse | 5′-TTAATGCATTTTTCTTGCTTCAATGTGAAGAATTCC-3′ |
| *lys-7* forward | 5′-GACACCAGCTCTTGCGTCAC-3′ |
| *lys-7* reverse | 5′-GATCGCTCTTGCAGTCAATC-3′ |
| *ctl-1* forward | 5′-TCGTTCATGCCAAGGGAGC-3′ |
| *ctl-1* reverse | 5′-GATCCCGATTCTCCAGCGAC-3′ |
| *ctl-2* forward | 5′-GAAGGTGTTGGATACCGGGG-3′ |
| *ctl-2* reverse | 5′-GGATGAGTGCCTTGACACGA-3′ |
| *gst-4* forward | 5′-CCCATTTTACAAGTCGATGG-3′ |
| *gst-4* reverse | 5′-CTTCCTCTGCAGTTTTTCCA-3′ |
| *hsp-16.1* forward | 5′-CCACTATTTCCGTCCAGCTC-3′ |
| *hsp-16.1* reverse | 5′-TGGAGAGCCTCTGCAAACTG-3′ |
| *hsp-16.2* forward | 5′-TATGGCTCTGATGGAACG-3′ |
| *hsp-16.2* reverse | 5′-GATTGATAGCGTACGACC-3′ |
| *dod-3* forward | 5′-GGAGTCCTGCTCTCAGATGAA-3′ |
| *dod-3* reverse | 5′-ACATGAACACCGGCTCATTC-3′ |
