# Supplementary material for: 20(S)-protopanaxadiol prolongs lifespan and enhances stress resistance in Caenorhabditis elegans via the insulin/IGF-1 signaling pathway
Source: Front Pharmacol. 2025 Oct 14;16:1657436. doi: 10.3389/fphar.2025.1657436 (PMC12558884; doi:10.3389/fphar.2025.1657436)
Supplement: Supplementary file 3 [file Table1.doc]

**Table S1. The primer sequences using for crossing**

| **Primers** | **Sequences of primers** |
| --- | --- |
| *daf-16* forward | 5′-ACATAGACGATTTCGAAAAGTTCAG-3′ |
| *daf-16* reverse | 5′-CATTAAGTGTCGAGTGAAGGGA-3′ |
| *daf-2* forward | 5′-ATGATTCATCAATGCGTACTCCTCA-3′ |
| *daf-2 reverse* | 5′-AGTGTTCAATGAGAGACGGACTGC-3′ |
