# Supplementary material for: 20(S)-protopanaxadiol prolongs lifespan and enhances stress resistance in Caenorhabditis elegans via the insulin/IGF-1 signaling pathway
Source: Front Pharmacol. 2025 Oct 14;16:1657436. doi: 10.3389/fphar.2025.1657436 (PMC12558884; doi:10.3389/fphar.2025.1657436)
Supplement: Supplementary file 4 [file Table2.docx]

**Table S2 Statistics of lifespan assays**

| **Strain** | **Fig.No.** | **Exp.No.** | **20(S)-PPD**  **(µM)** | **Mean lifespan± SD (days)** | **% Change**  **mean lifespan** | ***P value*** | **Total**  **animals Died/Total** |
| --- | --- | --- | --- | --- | --- | --- | --- |
| N2 | 1B-C | 1 | 0 | 23.92±5.18 | - | - | 48/48 |
|  |  | 2 |  | 24.37±5.10 | - | - | 51/51 |
|  |  | 3 |  | 23.04±5.90 | - | - | 56/62 |
|  |  | Total |  | 23.78±0.55 | - | - | 155/161 |
| N2 | 1B-C | 1 | 100 | 27.91±6.48 | 16.68 | <.0001^a^ | 44/44 |
|  |  | 2 |  | 28.74±4.86 | 17.93 | <.0001^a^ | 47/47 |
|  |  | 3 |  | 27.82±8.07 | 20.75 | <.0001^a^ | 55/59 |
|  |  | Total |  | 28.16±0.41 | 18.42 | <.0001^a^ | 146/150 |
| N2 | 1B-C | 1 | 200 | 29.13±5.88 | 21.78 | <.0001^a^ | 46/46 |
|  |  | 2 |  | 29.03±5.06 | 19.12 | <.0001^a^ | 39/39 |
|  |  | 3 |  | 28.92±7.07 | 25.52 | <.0001^a^ | 51/58 |
|  |  | Total |  | 29.03±0.09 | 22.08 | 0.0002^a^ | 136/143 |
| N2 | 1B-C | 1 | 400 | 27.89±5.82 | 16.60 | <.0001^a^ | 35/35 |
|  |  | 2 |  | 27.92±5.56 | 14.57 | <.0001^a^ | 47/47 |
|  |  | 3 |  | 25.74±7.27 | 11.72 | 0.007^a^ | 46/57 |
|  |  | Total |  | 27.18±1.02 | 14.30 | 0.0142^a^ | 128/139 |
| N2 | 1F | 1 | 0  (UV-killed OP50) | 24.13±7.93 | - | - | 48/50 |
|  |  | 2 |  | 24.09±7.33 | - | - | 46/50 |
|  |  | 3 |  | 24.10±7.46 | - | - | 48/50 |
|  |  | Total |  | 24.11±0.02 | - | - | 142/150 |
| N2 | 1F | 1 | 100 (UV-killed OP50) | 28.22±7.68 | 16.65 | 0.0014^b^ | 49/50 |
|  |  | 2 |  | 29.27±7.16 | 21.50 | 0.0002^b^ | 44/50 |
|  |  | 3 |  | 28.79±7.94 | 19.46 | 0.0002^b^ | 47/50 |
|  |  | Total |  | 28.76±0.43 | 19.41 | <.0001^b^ | 140/150 |
| N2 | 1F | 1 | 200 (UV-killed OP50) | 30.14±8.05 | 24.91 | <.0001^b^ | 49/50 |
|  |  | 2 |  | 29.90±7.99 | 24.12 | <.0001^b^ | 48/50 |
|  |  | 3 |  | 30.23±8.30 | 25.44 | <.0001^b^ | 47/50 |
|  |  | Total |  | 30.09±0.14 | 24.80 | <.0001^b^ | 144/150 |
| N2 | 1F | 1 | 400 (UV-killed OP50) | 28.85±8.49 | 19.56 | 0.0002^b^ | 47/52 |
|  |  | 2 |  | 28.94±8.30 | 20.13 | 0.0001^b^ | 48/50 |
|  |  | 3 |  | 28.32±8.19 | 17.56 | 0.0007^b^ | 47/50 |
|  |  | Total |  | 28.70±0.27 | 19.04 | <.0001^b^ | 142/152 |
| N2 | 5A-B | 1 | 0 | 22.52±6.31 | - | - | 50/50 |
|  |  | 2 |  | 22.70±6.15 | - | - | 46/50 |
|  |  | 3 |  | 22.21±6.15 | - | - | 48/50 |
|  |  | Total |  | 22.48±0.20 | - | - | 144/150 |
| N2 | 5A-B | 1 | 200 | 27.10±7.63 | 20.34 | 0.0001^c^ | 43/50 |
|  |  | 2 |  | 27.39±7.13 | 20.66 | <.0001^c^ | 49/50 |
|  |  | 3 |  | 27.66±7.27 | 24.54 | <.0001^c^ | 50/50 |
|  |  | Total |  | 27.38±0.23 | 21.80 | <.0001^c^ | 142/150 |
| *daf-2(e1368)* | 5A-B | 1 | 0 | 35.69±7.76 | 58.48 | <.0001^c^ | 51/55 |
|  |  | 2 |  | 34.18±10.21 | 50.57 | <.0001^c^ | 49/55 |
|  |  | 3 |  | 35.80±7.75 | 61.19 | <.0001^c^ | 51/55 |
|  |  | Total |  | 35.22±0.74 | 56.67 | <.0001^c^ | 151/165 |
| *daf-2(e1368)* | 5A-B | 1 | 200 | 32.08±10.12 | 42.54 | 0.1731^d^ | 51/55 |
|  |  | 2 |  | 32.69±9.80 | 44.01 | 0.1238^d^ | 52/60 |
|  |  | 3 |  | 35.20±8.73 | 58.49 | 0.9289^d^ | 50/60 |
|  |  | Total |  | 33.32±0.35 | 48.22 | 0.1558^d^ | 153/175 |
| *daf-2(e1370)* | 5A-B | 1 | 0 | 34.61±13.22 | 53.69 | <.0001^c^ | 46/60 |
|  |  | 2 |  | 35.54±12.61 | 56.56 | <.0001^c^ | 46/60 |
|  |  | 3 |  | 35.16±12.78 | 58.31 | <.0001^c^ | 45/55 |
|  |  | Total |  | 35.10±0.38 | 56.14 | <.0001^c^ | 137/175 |
| *daf-2(e1370)* | 5A-B | 1 | 200 | 37.52±12.73 | 66.61 | 0.3873^e^ | 49/60 |
|  |  | 2 |  | 36.06±12.36 | 58.85 | 0.8581^e^ | 49/60 |
|  |  | 3 |  | 35.57±12.02 | 60.15 | 0.7671^e^ | 46/55 |
|  |  | Total |  | 36.38±0.63 | 61.83 | 0.1181^e^ | 144/175 |
| N2 | 5C-F | 1 | 0 | 22.70±6.13 | - | - | 47/50 |
|  |  | 2 |  | 22.33±6.56 | - | - | 55/55 |
|  |  | 3 |  | 22.11±6.78 | - | - | 47/50 |
|  |  | Total |  | 22.38±0.25 | - | - | 149/155 |
| N2 | 5C-F | 1 | 200 | 27.41±8.58 | 20.75 | <.0001^f^ | 51/51 |
|  |  | 2 |  | 27.20±8.80 | 21.81 | <.0001^f^ | 49/50 |
|  |  | 3 |  | 28.18±7.52 | 27.45 | <.0001^f^ | 50/50 |
|  |  | Total |  | 27.60±0.42 | 23.32 | 0.0001^f^ | 150/151 |
| *eat-2*  *(ad1116)* | 5C-F | 1 | 0 | 27.02±7.17 | 19.03 | 0.0002^f^ | 46/50 |
|  |  | 2 |  | 27.38±7.00 | 22.62 | 0.0001^f^ | 45/50 |
|  |  | 3 |  | 27.68±6.46 | 25.19 | <.0001^f^ | 50/50 |
|  |  | Total |  | 27.36±0.27 | 22.25 | <.0001^f^ | 141/150 |
| *eat-2*  *(ad1116)* | 5C-F | 1 | 200 | 31.45±10.54 | 38.55 | 0.0011^g^ | 47/50 |
|  |  | 2 |  | 31.91±8.92 | 42.90 | 0.0013^g^ | 46/50 |
|  |  | 3 |  | 31.18±8.14 | 41.02 | 0.0049^g^ | 44/48 |
|  |  | Total |  | 31.51±0.30 | 40.80 | 0.0001^g^ | 137/148 |
| *isp-1(qm150)* | 5C-F | 1 | 0 | 25.06±8.32 | 10.40 | 0.0100^f^ | 50/50 |
|  |  | 2 |  | 25.40±7.70 | 13.75 | 0.0064^f^ | 48/50 |
|  |  | 3 |  | 25.83±7.27 | 16.82 | 0.0051^f^ | 47/50 |
|  |  | Total |  | 25.43±0.32 | 13.63 | 0.0004^f^ | 145/150 |
| *isp-1(qm150)* | 5C-F | 1 | 200 | 29.34±9.04 | 29.25 | 0.0077^h^ | 50/50 |
|  |  | 2 |  | 29.22±8.40 | 30.86 | 0.0042^h^ | 50/50 |
|  |  | 3 |  | 29.60±9.42 | 33.88 | 0.0026^h^ | 50/55 |
|  |  | Total |  | 29.39±0.16 | 31.32 | <.0001^h^ | 150/155 |
| *glp-1(e2141)* | 5C-F | 1 | 0 | 25.30±7.95 | 11.45 | 0.0096^f^ | 47/50 |
|  |  | 2 |  | 25.45±8.30 | 13.97 | 0.0042^f^ | 49/50 |
|  |  | 3 |  | 26.00±7.78 | 17.59 | 0.0026^f^ | 50/50 |
|  |  | Total |  | 25.58±0.30 | 14.30 | 0.0003^f^ | 146/150 |
| *glp-1(e2141)* | 5C-F | 1 | 200 | 29.22±10.58 | 28.72 | 0.0052^i^ | 49/50 |
|  |  | 2 |  | 30.76±9.03 | 37.75 | 0.0016^i^ | 46/50 |
|  |  | 3 |  | 29.67±9.36 | 34.19 | 0.0075^i^ | 49/50 |
|  |  | Total |  | 29.89±0.65 | 33.56 | 0.0010^i^ | 144/50 |
| N2 | 6A | 1 | 0 | 21.80±7.01 | - | - | 46/50 |
|  |  | 2 |  | 23.03±4.17 | - | - | 53/55 |
|  |  | 3 |  | 23.07±5.72 | - | - | 43/50 |
|  |  | Total |  | 22.64±0.59 | - | - | 142/155 |
| N2 | 6A | 1 | 200 | 27.52±5.30 | 26.24 | <.0001^j^ | 46/50 |
|  |  | 2 |  | 28.24±8.21 | 22.62 | <.0001^j^ | 50/50 |
|  |  | 3 |  | 28.89±5.93 | 25.23 | <.0001^j^ | 44/45 |
|  |  | Total |  | 28.22±0.56 | 24.65 | 0.0006^j^ | 140/145 |
| *daf-16(mu86)* | 6A | 1 | 0 | 19.04±4.31 | -12.66 | 0.0003^j^ | 46/52 |
|  |  | 2 |  | 18.08±4.47 | -21.49 | <.0001^j^ | 48/50 |
|  |  | 3 |  | 19.45±4.29 | -15.69 | <.0001^j^ | 51/54 |
|  |  | Total |  | 18.56±0.57 | -18.02 | 0.0029^j^ | 145/156 |
| *daf-16(mu86)* | 6A | 1 | 200 | 18.25±4.40 | -16.28 | 0.3529^k^ | 49/54 |
|  |  | 2 |  | 17.19±4.70 | -25.36 | 0.5474^k^ | 47/50 |
|  |  | 3 |  | 19.71±3.89 | -14.56 | 0.8641^k^ | 55/55 |
|  |  | Total |  | 18.38±1.03 | -18.82 | 0.6014^k^ | 151/159 |
| N2 | 6B | 1 | 0 | 22.27±6.60 | - | - | 60/65 |
|  |  | 2 |  | 22.23±6.45 | - | - | 60/60 |
|  |  | 3 |  | 22.38±6.22 | - | - | 61/65 |
|  |  | Total |  | 22.29±0.06 | - | - | 181/190 |
| N2 | 6B | 1 | 200 | 27.19±7.56 | 22.09 | <.0001^l^ | 59/60 |
|  |  | 2 |  | 27.59±8.76 | 24.11 | <.0001^l^ | 59/60 |
|  |  | 3 |  | 28.08±7.76 | 25.47 | <.0001^l^ | 60/60 |
|  |  | Total |  | 27.62±0.36 | 23.91 | <.0001^l^ | 178/180 |
| *daf-2(e1370);daf-16(mu86)* | 6B | 1 | 0 | 16.92±6.82 | -24.02 | <.0001^l^ | 61/61 |
|  |  | 2 |  | 15.83±6.72 | -28.79 | <.0001^l^ | 60/60 |
|  |  | 3 |  | 17.06±6.51 | -23.77 | <.0001^l^ | 52/60 |
|  |  | Total |  | 16.60±0.55 | -25.53 | 0.0001^l^ | 173/181 |
| *daf-2(e1370);daf-16(mu86)* | 6B | 1 | 200 | 17.73±6.89 | -20.39 | 0.3656^m^ | 60/65 |
|  |  | 2 |  | 16.61±7.13 | -25.28 | 0.3782^m^ | 57/60 |
|  |  | 3 |  | 16.62±6.82 | -25.74 | 0.8582^m^ | 61/61 |
|  |  | Total |  | 16.99±0.53 | -23.78 | 0.5154^m^ | 178/186 |

This table shows the lifespan results of Fig. 1B-C, 1F, 5A-F and 6A-B. The standard error represented by SD is the standard deviation of the sample mean. Total represents the total number of nematodes, that is, the number of dead animals plus the number of deleted nematodes (nematodes that crawled out of the culture dish, those that caused wormbags, and those whose bodies burst are considered deleted nematodes and are not included in the statistics). The *p* value is calculated as follows: ^a^N2 (Fig. 1B-C), ^b^N2 (UV-killed OP50) (Fig. 1F), ^c^N2 (Fig. 5A-B), ^d^*daf-2(e1368)* (Fig. 5A-B), ^e^*daf-2(e1370)* (Fig. 5A-B), ^f^N2 (Fig. 5C-F), ^g^*eat-2(ad1116)* (Fig. 5C-F), ^h^*isp-1(qm150)* (Fig. 5C-F), ^i^*glp-1(e2141)* (Fig. 5C-F), ^j^N2 (Fig. 6A), ^k^*daf-16(mu86)* (Fig. 6A), ^l^N2 (Fig. 6B), ^m^*daf-2(e1370);daf-16(mu86)* (Fig. 6B). All the statistical data were analyzed using Graphpad Prism 8 software. The statistical data were all tested using log-rank (Mantel-Cox).
